# Supplementary figures and images for: The structure of MgtE in the absence of magnesium provides new insights into channel gating
Source: PLoS Biol. 2021 Apr 27;19(4):e3001231. doi: 10.1371/journal.pbio.3001231 (PMC8104411; doi:10.1371/journal.pbio.3001231)

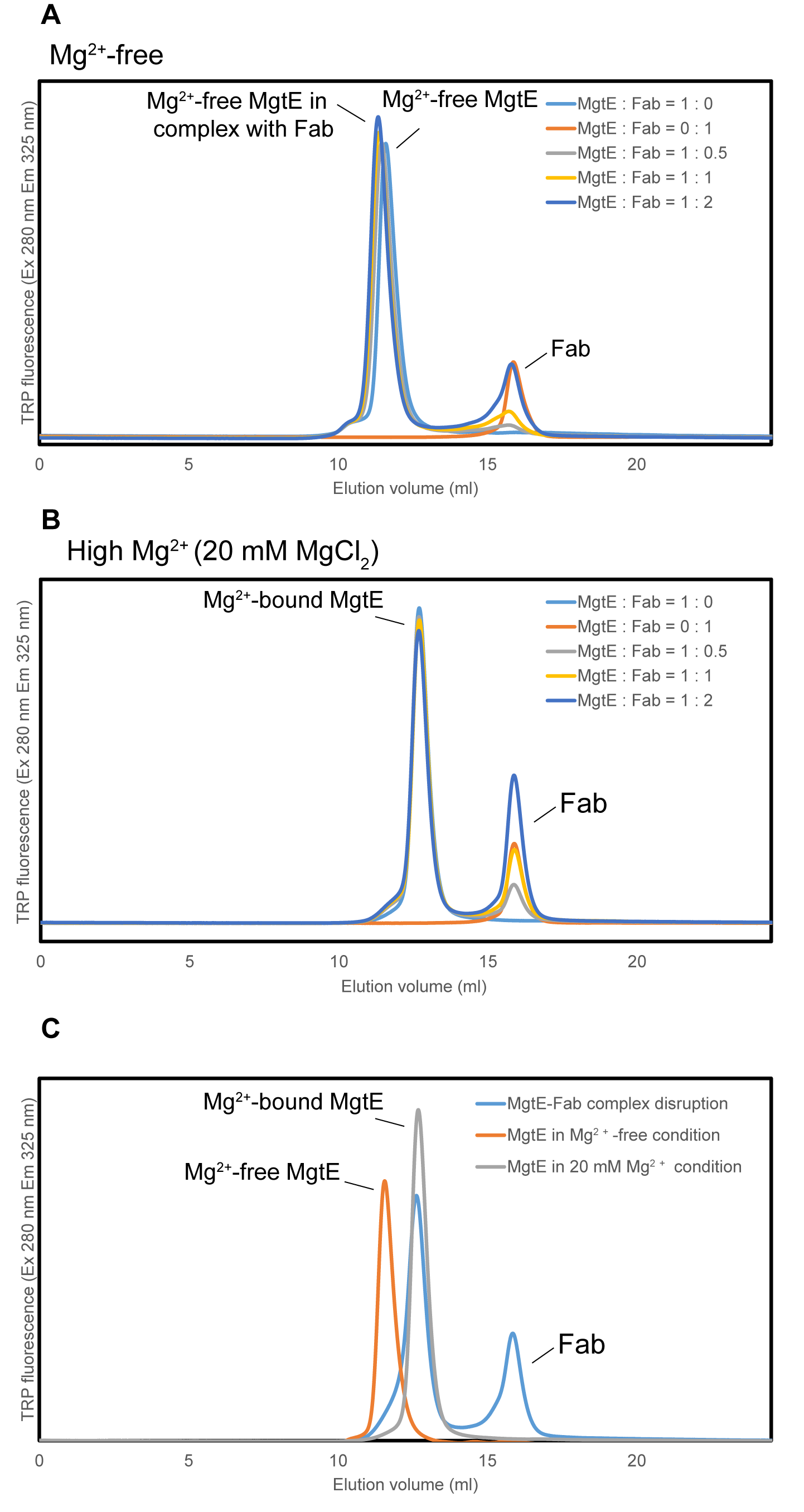

Supplement: S1 Fig — (A, B) FSEC analysis of MgtE-Fab complex formation. The MgtE-Fab complex was formed by adding Fab to MgtE in 0 mM MgCl2 (A) and in 20 mM MgCl2 (B) at the indicated mass ratios. The FSEC running buffer included 0 mM for (A) and 20 mM MgCl2 for (B). (C) The complex disruption experiment was performed by adding Mg2+ at a final concentration of 20 mM to the preformed MgtE-Fab complex, which was prepared by mixing MgtE and Fab705 at a mass ratio of 1:2 in 0 mM Mg2+. In addition to the FSEC profile for the complex disrupted sample (blue), the FSEC profiles of Fab-free MgtE in Mg2+-free conditions (orange) and 20 mM MgCl2 (gray) are also shown. The FSEC running buffer includes 20 mM MgCl2, except for the FSEC of MgtE in Mg2+-free conditions. In FSEC profiles, Mg2+-free MgtE (A, C) eluted earlier than Mg2+-bound MgtE (B, C), probably because the conformation of MgtE, in particular the conformation of the cytoplasmic domain of MgtE, is more compact in the presence of Mg2+ than in the absence of Mg2+ as shown in Fig 1B. FSEC, fluorescence detection size exclusion chromatography. (TIF) [file pbio.3001231.s001.tif]

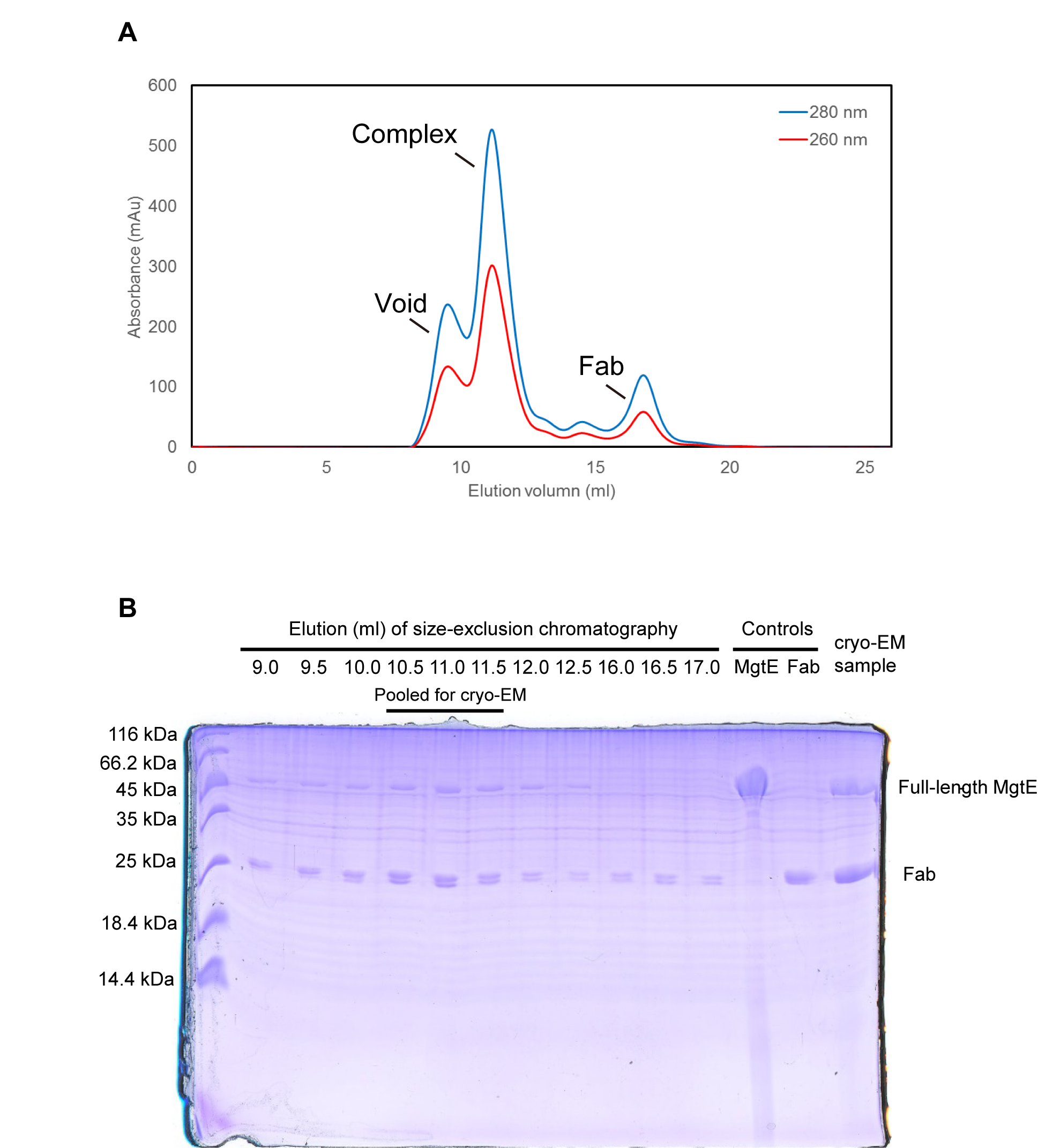

Supplement: S2 Fig — (A) SEC of the MgtE-Fab complex. The former, middle, and latter peaks were the void, Fab-MgtE complex, and free Fab, respectively. The fractions from the 10.5 to 12.0 ml elution positions were pooled as the cryo-EM sample. (B) SDS-PAGE of the SEC fractions and the cryo-EM sample. The individual numerical values that underlie the summary data displayed in this figure can be found in S1 Data. cryo-EM, cryo-electron microscopy; SDS-PAGE, sodium dodecyl sulfate-polyacrylamide gel electrophoresis; SEC, size exclusion chromatography. (TIF) [file pbio.3001231.s002.tif]

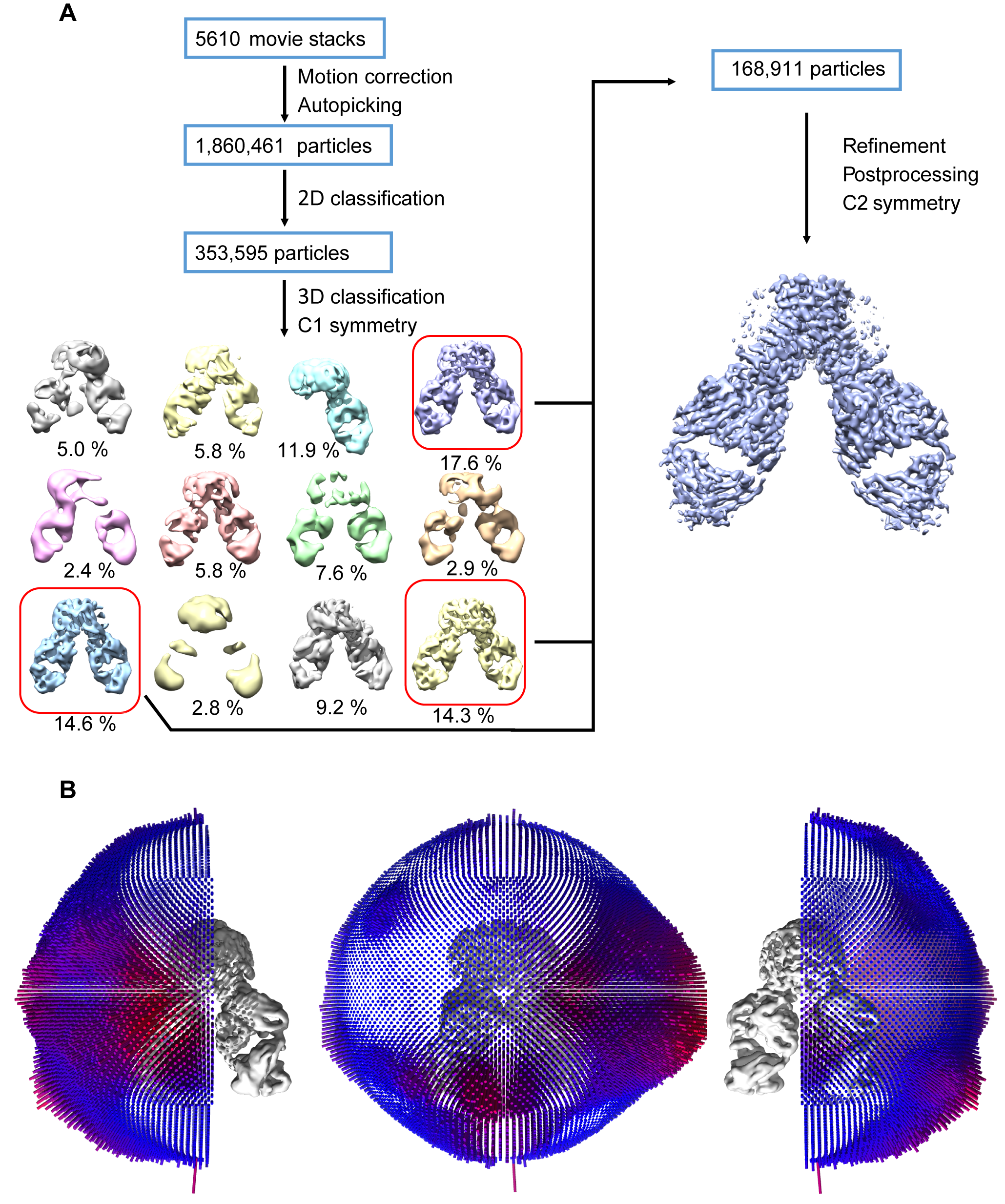

Supplement: S3 Fig — (A) Overview of the data processing workflow, including particle picking, classification, and 3D refinement. All processing steps were performed in RELION. (B) Euler angle distribution plot of all particles included in the calculation of the MgtE-Fab complex, with C2 symmetry imposed. cryo-EM, cryo-electron microscopy. (TIF) [file pbio.3001231.s003.tif]

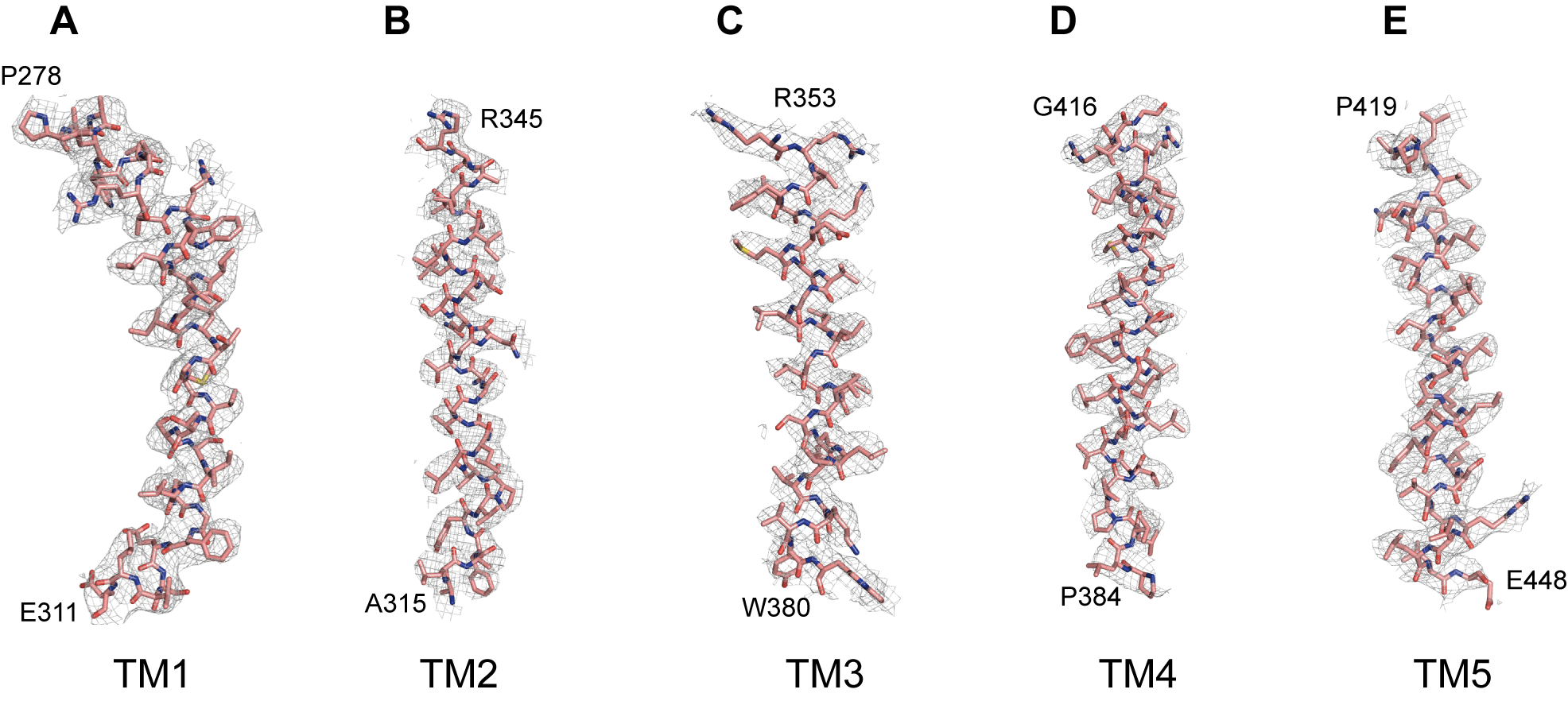

Supplement: S4 Fig — (A–E) Representative EM density maps contoured at 4.0 σ. The TM1 helix (residues 278–311) (A), TM2 helix (residues 315–345) (B), TM3 helix (residues 353–380) (C), TM4 helix (residues 384–416) (D), and TM5 helix (residues 419–448) (E) in chain A are shown as stick representations. EM, electron microscopy; TM, transmembrane. (TIF) [file pbio.3001231.s004.tif]

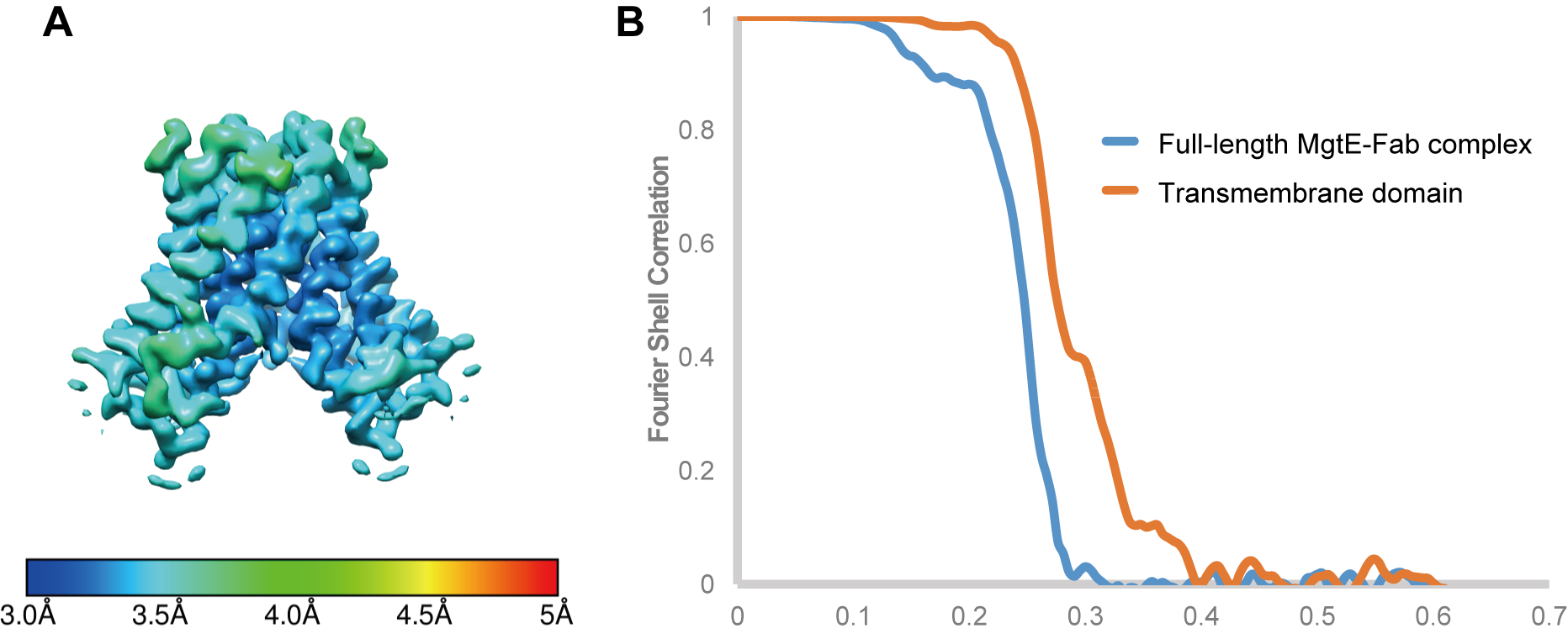

Supplement: S5 Fig — (A) Side view of the EM density map for the TM domain obtained by signal subtraction, colored according to local resolution, calculated using RELION. (B) Gold standard FSC for estimating resolution. The individual numerical values that underlie the summary data displayed in this figure can be found in S1 Data. EM, electron microscopy; FSC, Fourier shell correlation; TM, transmembrane. (TIF) [file pbio.3001231.s005.tif]

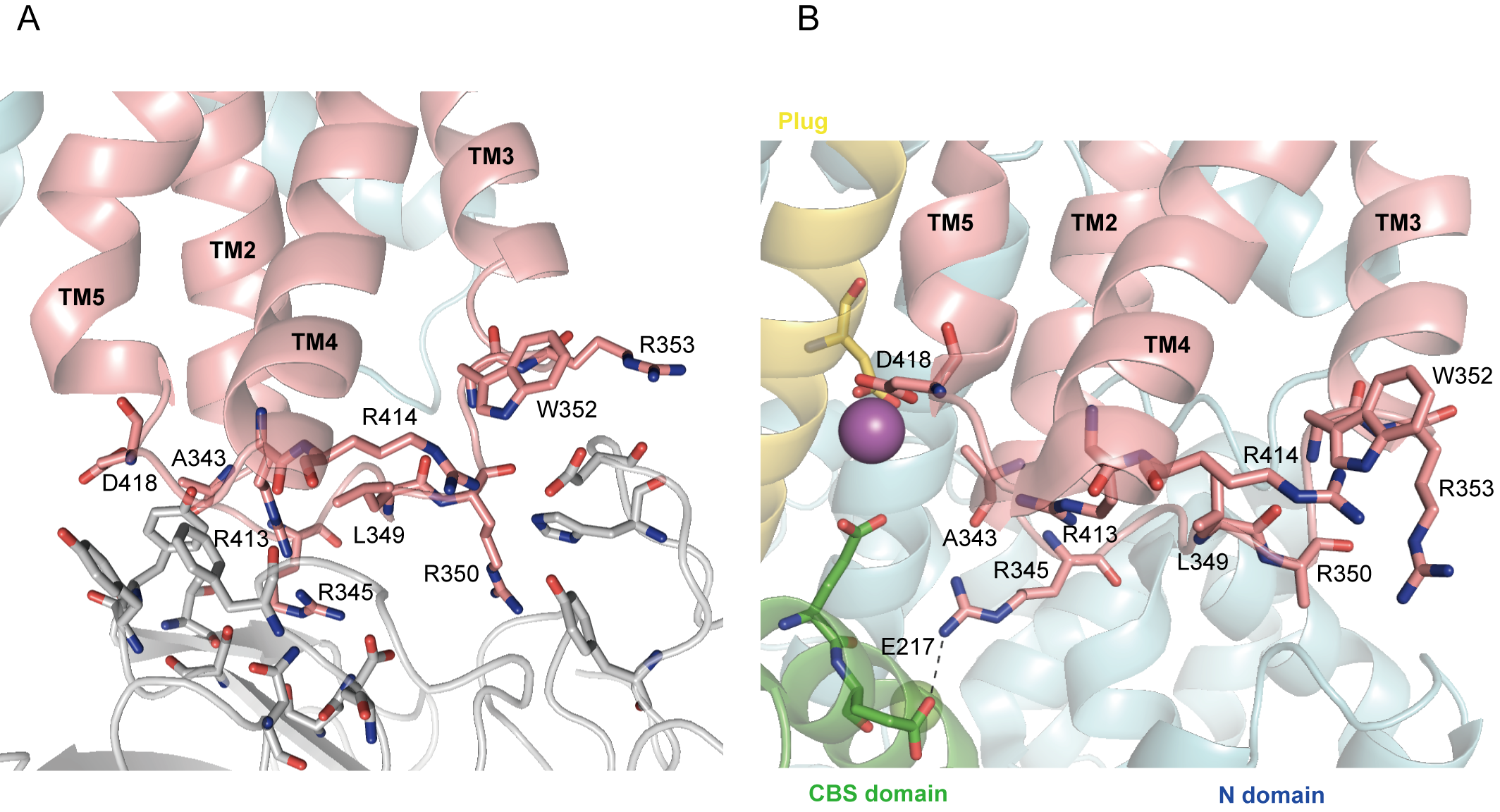

Supplement: S6 Fig — A close-up view of the MgtE-Fab interface on the cytoplasmic side (A) and of the corresponding region in full-length MgtE in the Mg2+-bound form (PDB ID: 2ZY9) (B). The coloring scheme of MgtE is the same as that in Fig 1, and Fab is colored gray. Residues located at the MgtE-Fab interface are depicted as stick representations. Mg2+ ions are depicted as purple spheres. (TIF) [file pbio.3001231.s006.tif]

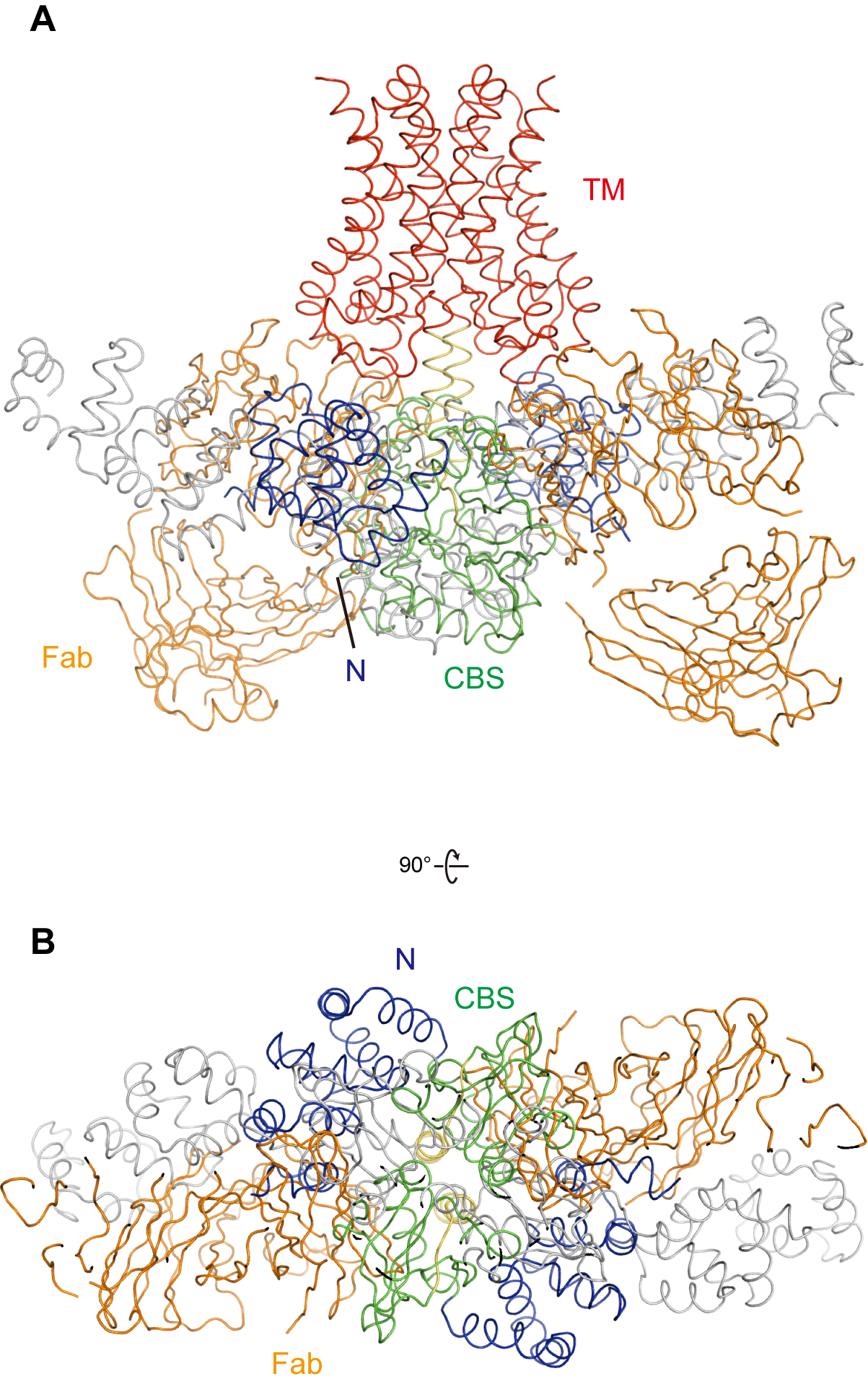

Supplement: S7 Fig — Superimpositions of our cryo-EM structure of the MgtE-Fab complex under Mg2+-free conditions and the Mg2+-free cytoplasmic domain structure (PDB ID: 2YVZ) onto full-length MgtE in the Mg2+-bound form (PDB ID: 2ZY9), viewed parallel to the membrane (A) and from the cytoplasmic side (B). The coloring scheme of the full-length MgtE structure is the same as that in Fig 1. The Mg2+-free cytoplasmic domain structure is colored gray. The TM domain and Fabs in the MgtE-Fab complex are colored red and orange, respectively. cryo-EM, cryo-electron microscopy; TM, transmembrane. (TIF) [file pbio.3001231.s007.tif]

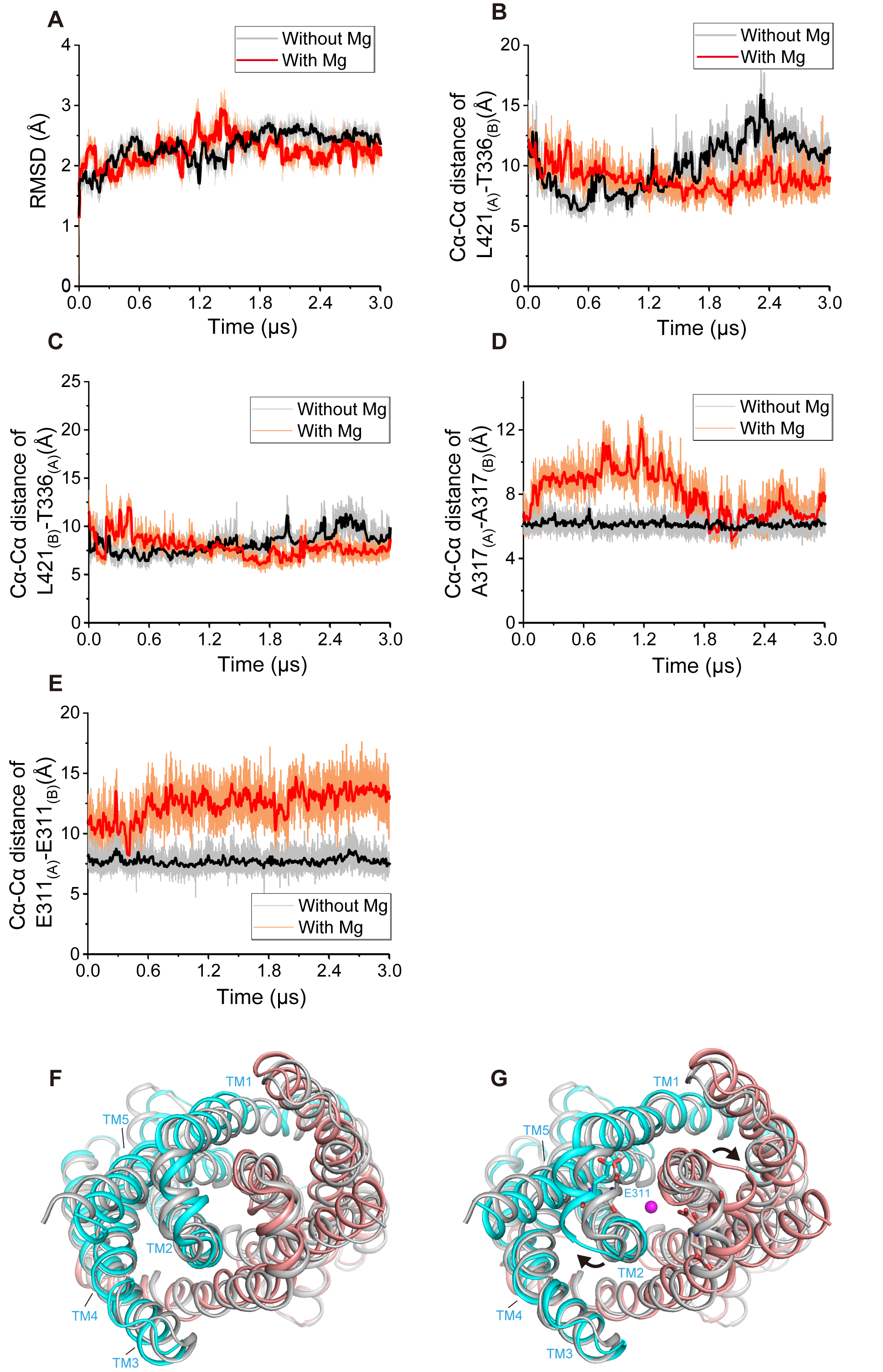

Supplement: S8 Fig — (A) Structural deviations from the Mg2+-free MgtE TM domain structure during the 3-μs MD simulations. (B–E) Cα distances between Thr336 (chain B) and Leu421 (chain A) (B), between Thr336 (chain A) and Leu421 (chain B) (C), between Ala317 (chains A and B) (D), and between Glu311 (chains A and B) (E) during the 3-μs MD simulation. The MD simulations were performed with (red) and without (black) adding Mg2+ ion at the Mg2+ binding site in the pore. The position of the added Mg2+ ion in the pore was based on the previous Mg2+-bound MgtE structure (PDB ID: 2ZY9) (Fig 1A). (F, G) The Mg2+-free MgtE TM domain structures after the 3-μs MD simulations with (G) and without (F) adding Mg2+ ion in the pore are superposed onto Mg2+-bound MgtE in the closed state (PDB ID:2ZY9) using the Cα positions of the TM domain dimer. The MgtE structures after the 3-μs MD simulations are colored salmon (chain A) and cyan (chain B). Mg2+-bound MgtE is colored gray. Mg2+ ions are shown as purple spheres. The black arrows indicate the structural changes. The individual numerical values that underlie the summary data displayed in this figure can be found in S1 Data. MD, molecular dynamics; TM, transmembrane. (TIF) [file pbio.3001231.s008.tif]

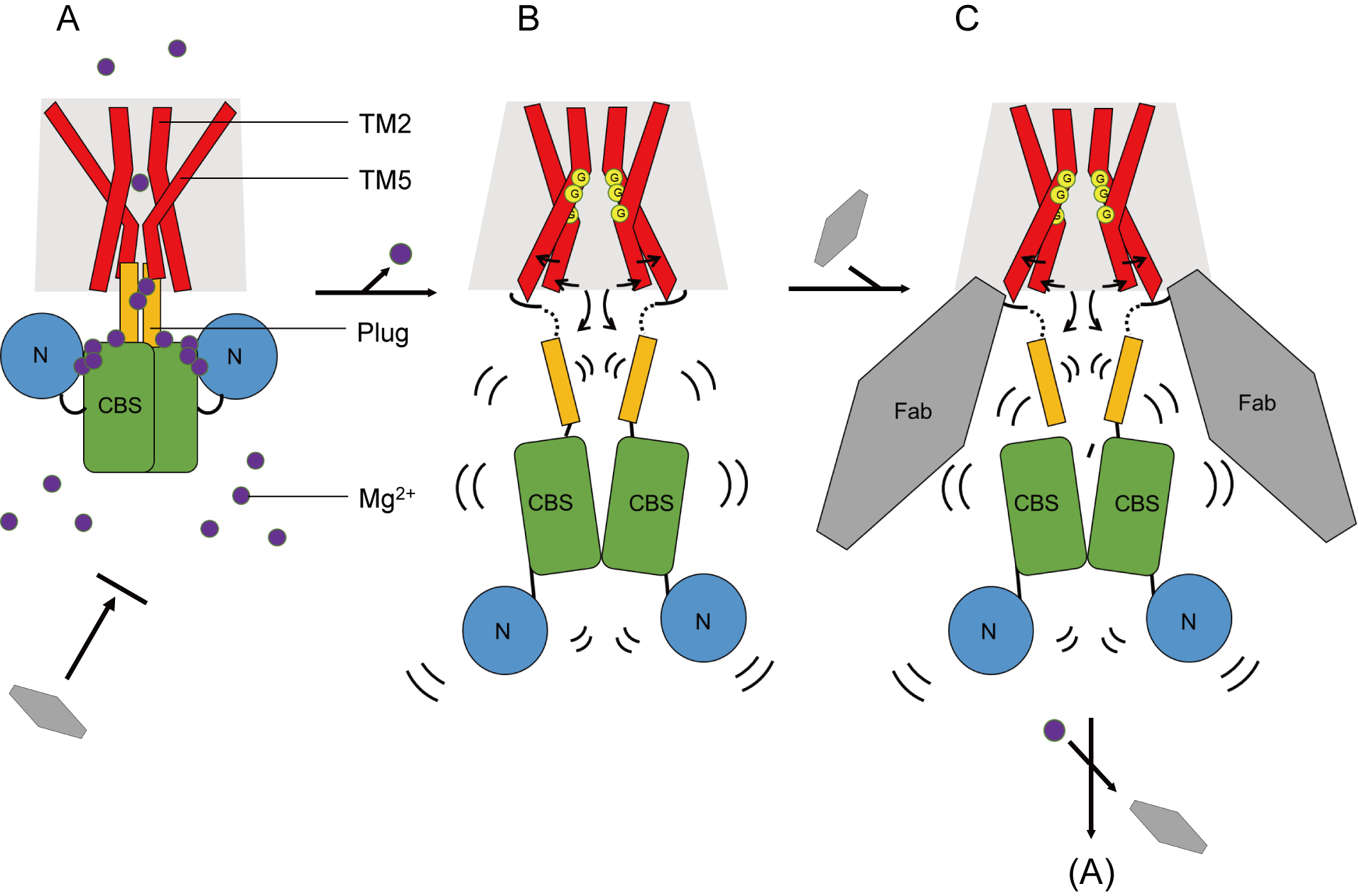

Supplement: S9 Fig — A cartoon of the proposed effect of Fab705 in the presence of Mg2+ (A) and under Mg2+-free conditions (B, C). Briefly, Fab705 cannot bind to MgtE at high Mg2+ concentrations (A). At low Mg2+ concentrations, Fab705 can bind to MgtE but does not either positively or negatively modulate channel opening, and a high concentration of Mg2+ ions can still close the MgtE channel by disrupting the MgtE-Fab complex (C). (TIF) [file pbio.3001231.s009.tif]

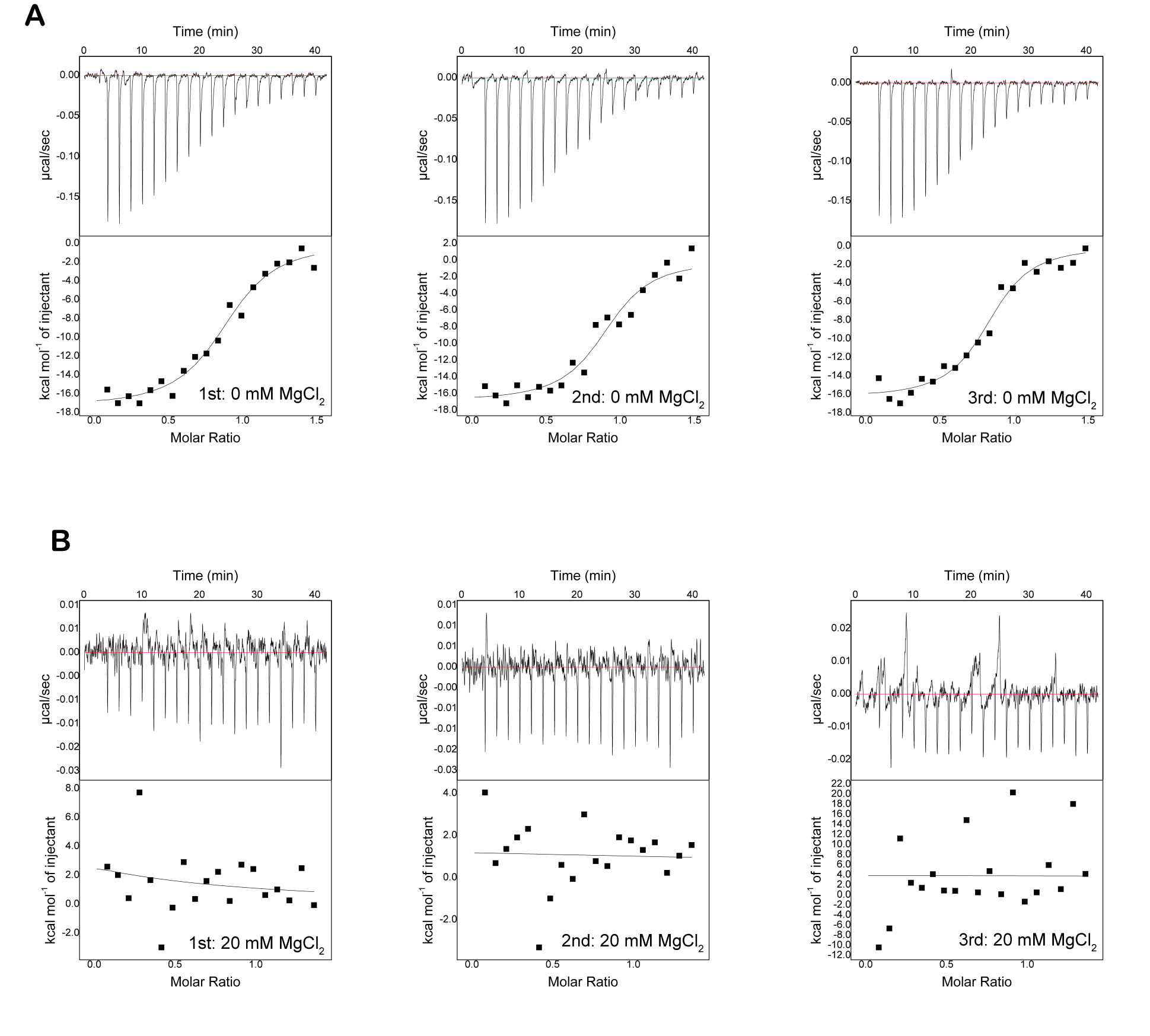

Supplement: S10 Fig — (A, B) ITC profiles of MgtE with Fab705 in the presence of 0 mM MgCl2 (A) and 20 mM MgCl2 (B). The individual numerical values that underlie the summary data displayed in this figure can be found in S1 Data. ITC, isothermal titration calorimetry. (TIF) [file pbio.3001231.s010.tif]

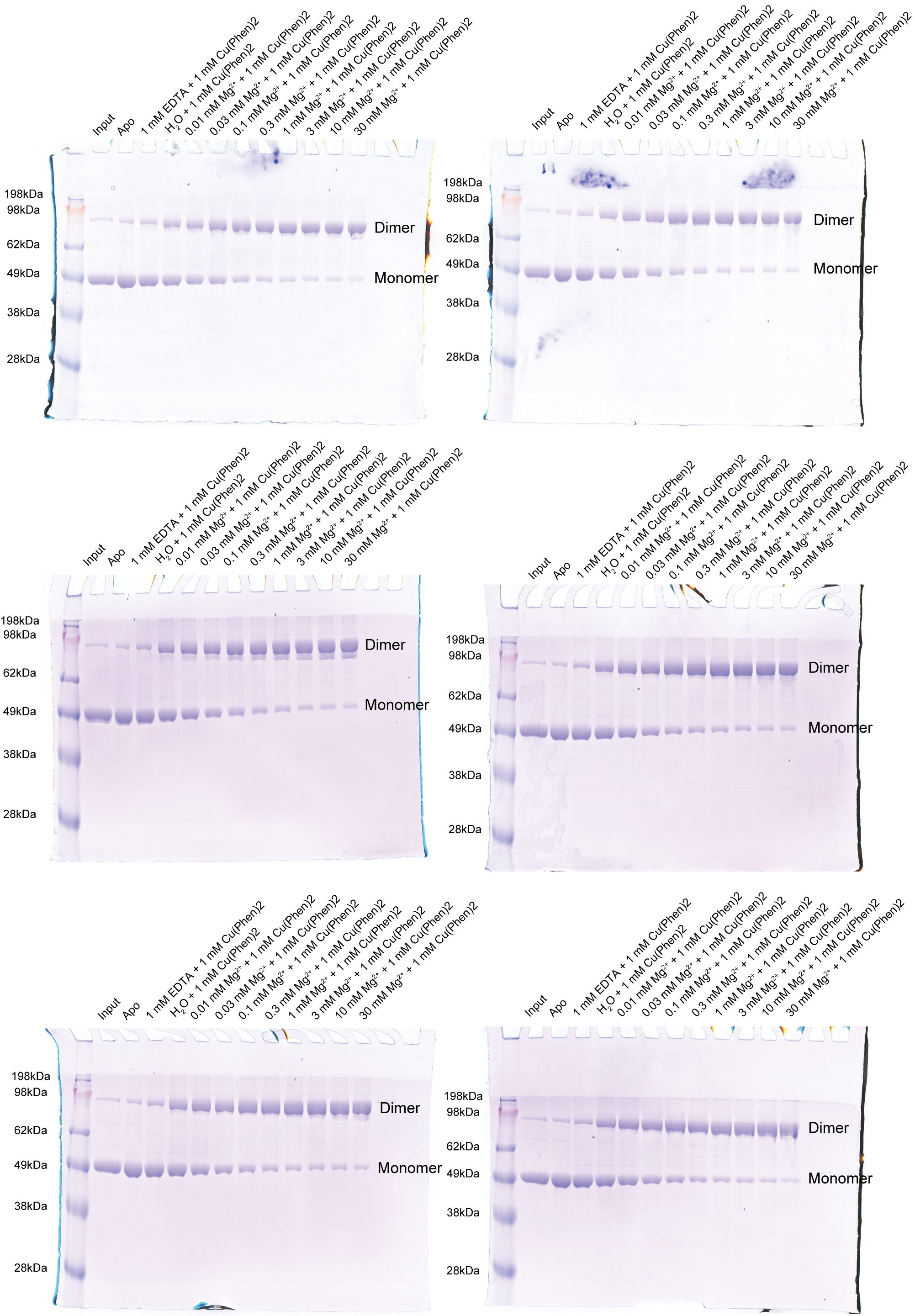

Supplement: S11 Fig — SDS-PAGE, sodium dodecyl sulfate-polyacrylamide gel electrophoresis. (TIF) [file pbio.3001231.s011.tif]

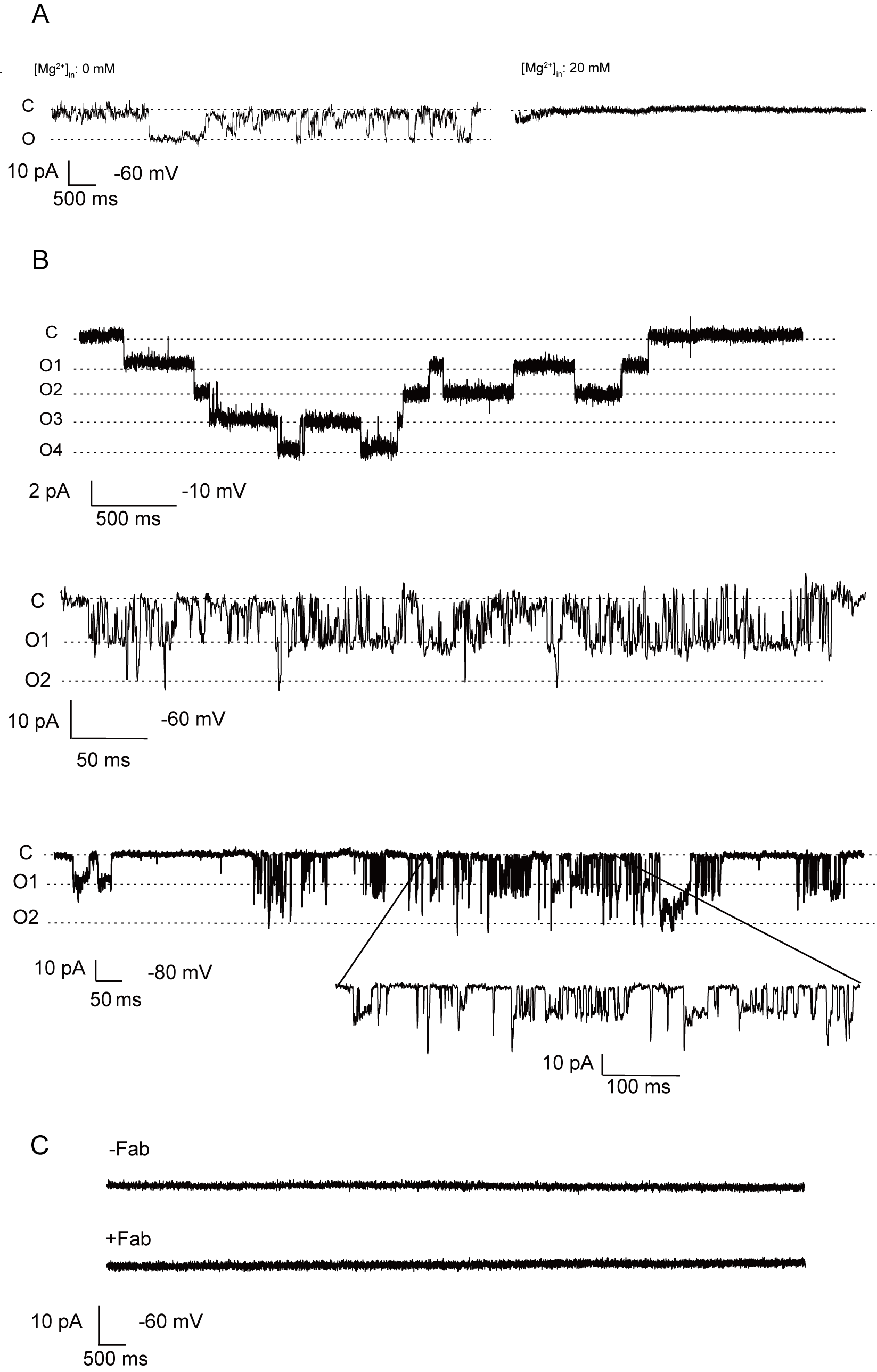

Supplement: S12 Fig — (A) Representative current traces from the same patch of triple-knockout E. coli spheroplasts expressing MgtE under 0 mM MgCl2 (left) and under 20 mM MgCl2 (right) in the bath solution. Application of the bath solution containing 20 mM MgCl2 inhibited channel opening. Experiments were repeated 3 times, and similar results were obtained. (B) Representative current traces of triple-knockout E. coli spheroplasts expressing MgtE with multiple channel openings in the patch. Of the 18 patches, 14 of them showed channel currents, and 4 contained 2–4 channels. (C) Representative current traces from triple-knockout E. coli spheroplasts harboring the empty vector. Experiments were repeated 7 times, and no channel currents were obtained. The individual numerical values that underlie the summary data displayed in this figure can be found in S1 Data. (TIF) [file pbio.3001231.s012.tif]

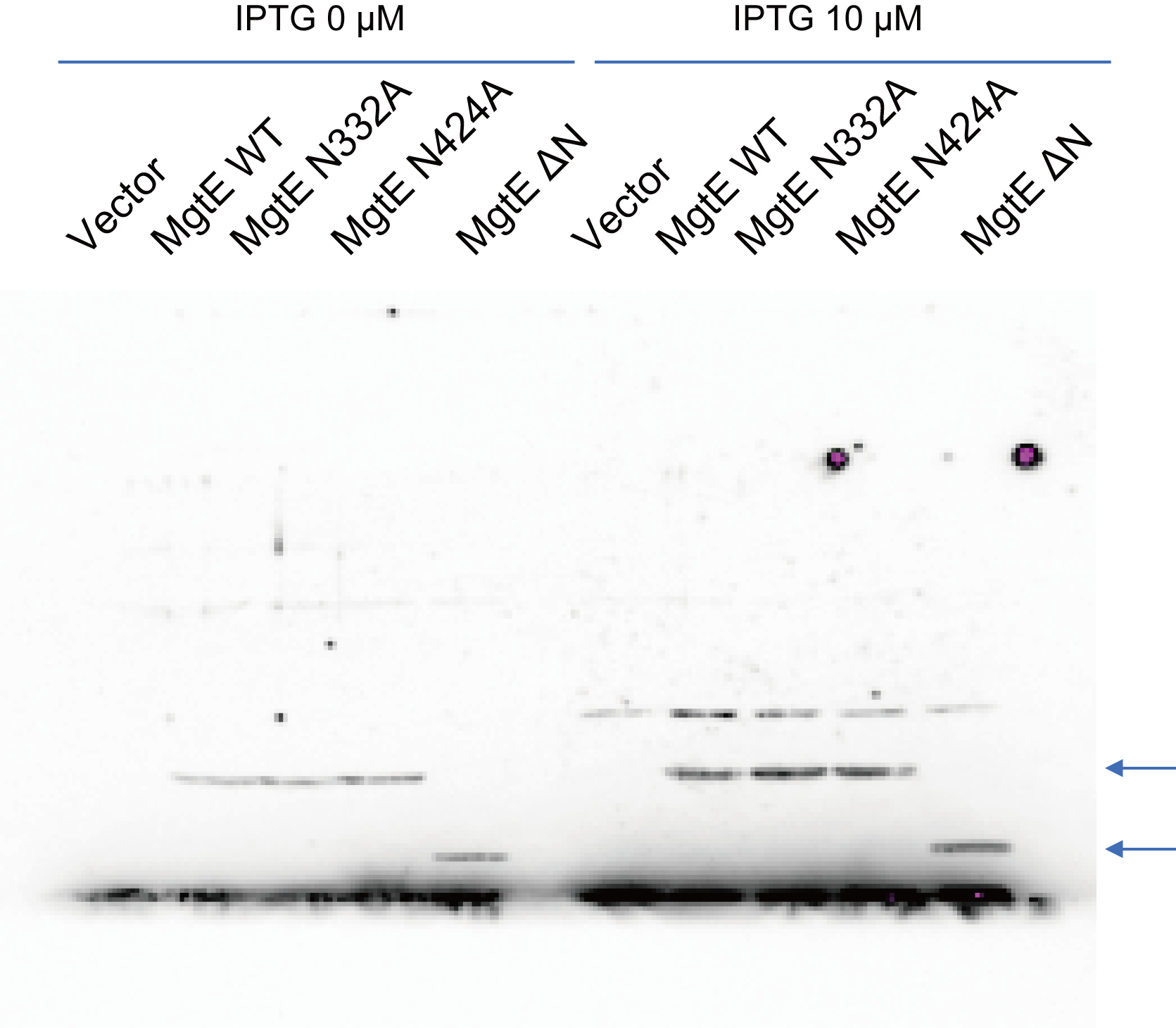

Supplement: S13 Fig — (TIF) [file pbio.3001231.s013.tif]

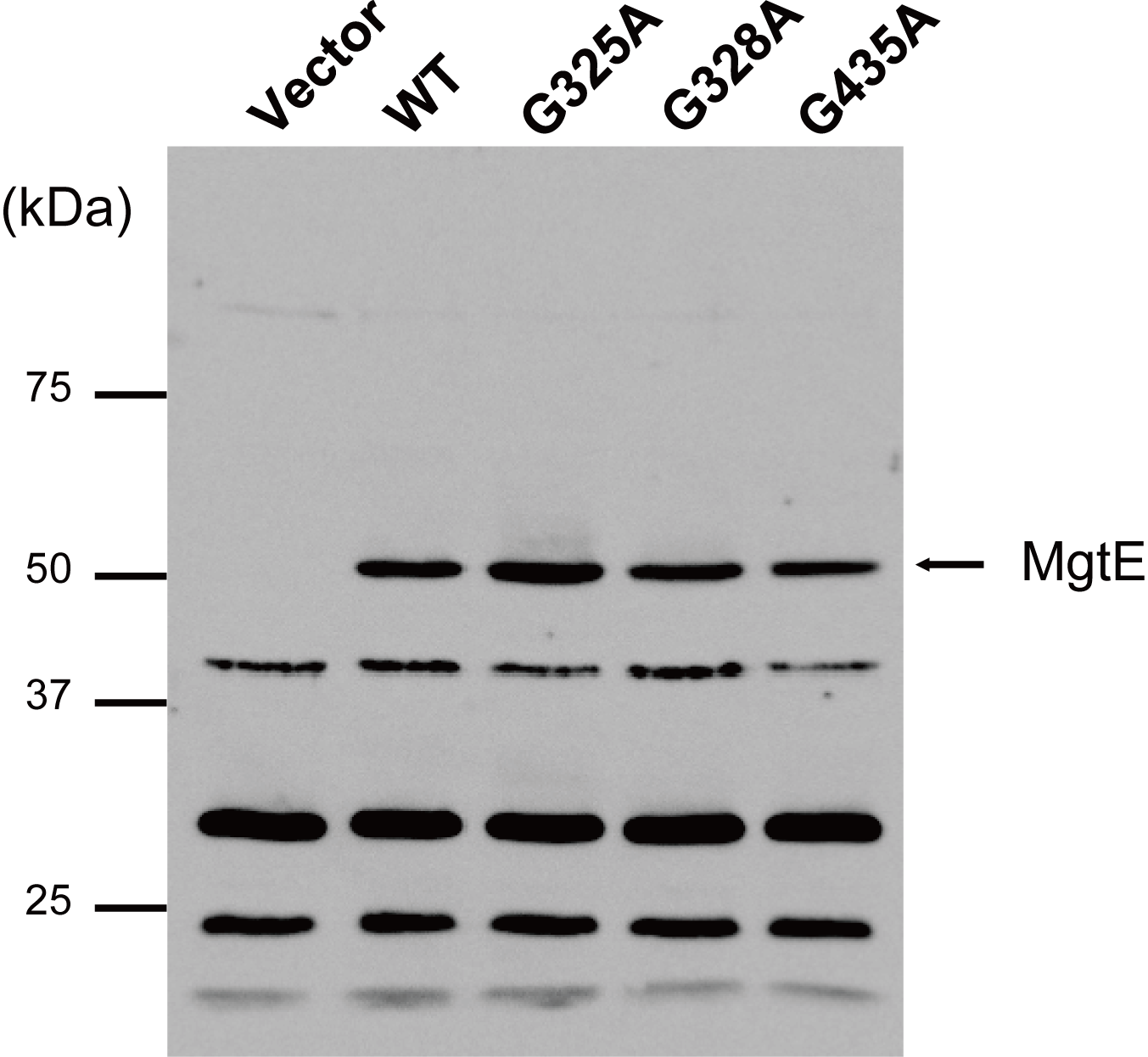

Supplement: S14 Fig — Uncropped western blots are provided in S1 Raw Images. (TIF) [file pbio.3001231.s014.tif]

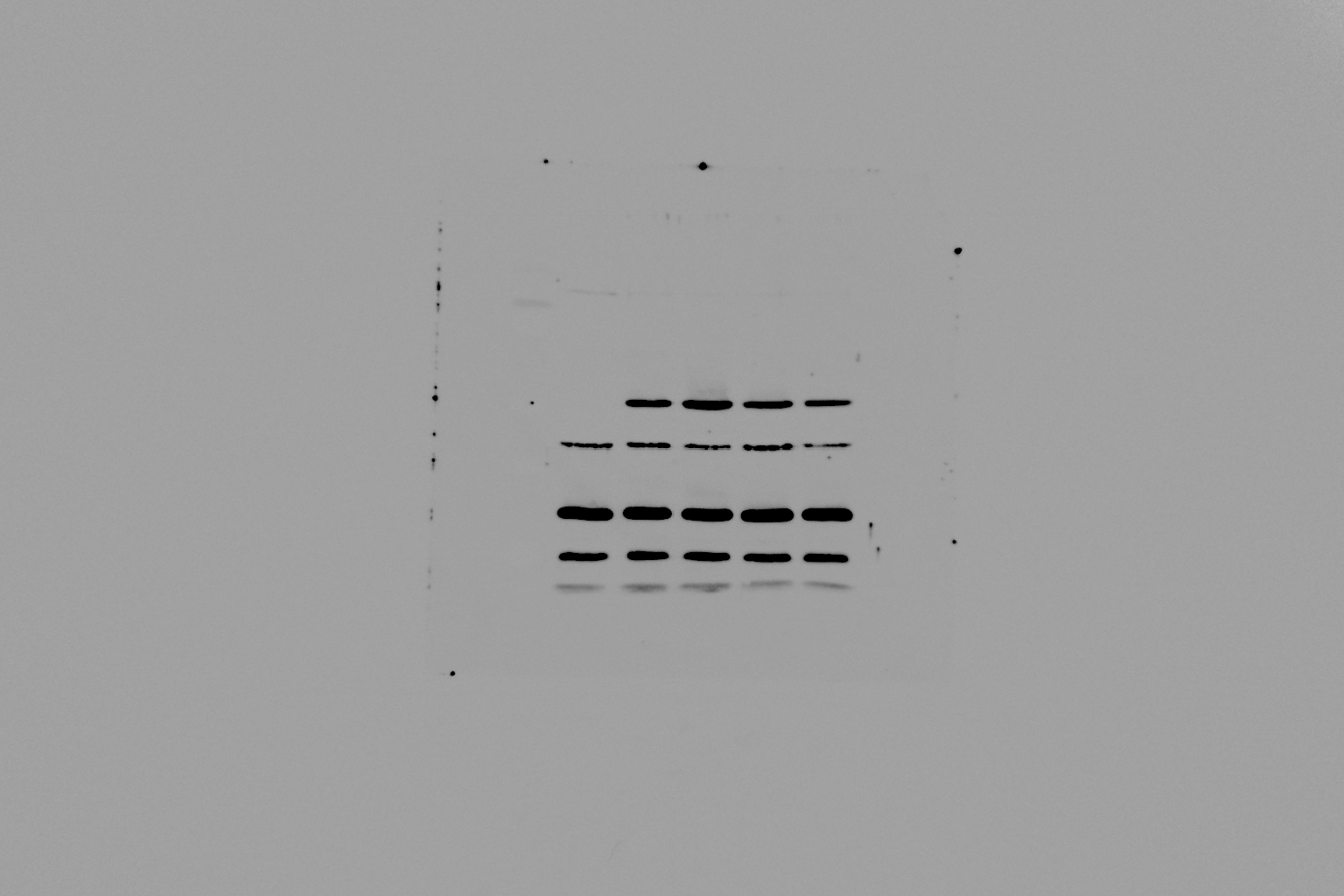

Supplement: S1 Raw Images — (TIF) [file pbio.3001231.s019.tif]
