# Supplementary material for: The structure of MgtE in the absence of magnesium provides new insights into channel gating
Source: PLoS Biol. 2021 Apr 27;19(4):e3001231. doi: 10.1371/journal.pbio.3001231 (PMC8104411; doi:10.1371/journal.pbio.3001231)
Supplement: S1 Table — ITC, isothermal titration calorimetry. (PDF) [file pbio.3001231.s016.pdf]

	N	ΔH° (kcal/mol)	−TΔS° (kcal/mol)	ΔG° (kcal/mol)	K_D (nM)
1st:Without Mg^{2+}	0.89 ± 0.02	-17.35 ± 0.56	8.23	-9.12 ± 0.56	216.1 ± 52.7
2nd:Without Mg^{2+}	0.90 ± 0.03	-16.91 ± 0.78	7.63	-9.28 ± 0.78	186.8 ± 72.4
3rd: Without Mg^{2+}	0.83 ± 0.02	-16.35 ± 0.57	7.04	-9.31± 0.57	163.8 ± 46.4
