# Supplementary material for: The structure of MgtE in the absence of magnesium provides new insights into channel gating
Source: PLoS Biol. 2021 Apr 27;19(4):e3001231. doi: 10.1371/journal.pbio.3001231 (PMC8104411; doi:10.1371/journal.pbio.3001231)
Supplement: S2 Table — cryo-EM, cryo-electron microscopy. (PDF) [file pbio.3001231.s017.pdf]

	MgtE-Fab (EMDB-0869) (PDB 6LBH)
**Data collection and processing**	
Magnification	29,000x
Voltage (kV)	300
Electron exposure (e–/Å^2)	70
Defocus range (μm)	-1.5 to -2.3
Pixel size (Å)	0.82
Symmetry imposed	*C*2
Initial particle images (no.)	1,860,461
Final particle images (no.)	168,911
Map resolution (Å)	3.7
FSC threshold	0.143
Map resolution range (Å)	2.84-4.5
**Refinement**	
Initial model used (PDB code)	This study
Model resolution (Å)	3.7
FSC threshold	0.143
Model resolution range (Å)	2.84-4.5
Map sharpening *B* factor (Å^2)	-167
Model composition	
Non-hydrogen atoms	9,234
Protein residues	1,236
Ligands	NA
B factors (Å^2)	
Protein	55.87
Ligand	NA
R.m.s. deviations	
Bond lengths (Å)	0.010
Bond angles (°)	1.069
Validation	
MolProbity score	2.26
Clashscore	9.97
Poor rotamers (%)	2.37
Ramachandran plot	
Favored (%)	92.9
Allowed (%)	6.9
Disallowed (%)	0.2
