# Supplementary material for: The structure of MgtE in the absence of magnesium provides new insights into channel gating
Source: PLoS Biol. 2021 Apr 27;19(4):e3001231. doi: 10.1371/journal.pbio.3001231 (PMC8104411; doi:10.1371/journal.pbio.3001231)
Supplement: Attachment — Submitted filename: 110112_1_rebuttal_1913662_qjml87.docx [file pbio.3001231.s020.docx]

**Reviewer #1 (Evidence, reproducibility and clarity (Required)):**

***“The manuscript by Jin and coworkers reports the cryo-EM structure of the Thermus thermophilus MgtE magnesium ion channel in Mg2+ free conditions bound to an antibody Fab, Fab705. The structure shows a complex in which each subunit of the channel dimer is bound by one Fab705. The conformation shows an open intracellular side, but also shows that the channel remains closed on the extracellular side. The cytoplasmic assembly that is stabilized by Mg2+ is invisible and it is inferred that it has become mobile. Further, the binding of Fab705 to the intracellular side of the transmembrane domains is clearly structurally incompatible with the positions of the intracellular domains in the closed channel state. The authors use crosslinking and functional studies to validate key interactions and conformational changes inferred from this structure relative to the closed MtgE. Overall, the data are mostly convincing but there are a few issues that must be addressed.”***

We appreciate the positive response from Reviewer #1. We have addressed the specific comments below.

***“**Major comments:*****

***“The stoichiometry for the ITC is substantially different from the expected 1:1. This is likely due to errors in determining the concentration of the components. It is not stated in the methods how these were measured and the use of symbols indicting approximate values indicates that these experiments were not done with the accuracy required for meaningful analysis.”***

We appreciate this comment. After checking the experimental records, we realized our simple mistake in the Fab concentration value we used in the ITC data analysis. The calculated absorbance values at 280 nm for 1 mg/ml are 2.071 and 1.42 for the Fab heavy and light chains, respectively, but we mistakenly applied 1.0 for both when we analysed the ITC data. After correcting this, the stoichiometry was nearly 1:1, and the *K*_d_ value was still consistent with the previous value (**Cover Letter Table 1**). Notably, we did not find this type of mistake in other experiments. We apologize for our carelessness, and we will correct the figure, table and associated description based on the corrected ITC analysis in the revised manuscript.

|  | N | ΔΗ° (kcal/mol) | −TΔS° (kcal/mol) | ΔG° (kcal/mol) | K_D_ (nM) |
| --- | --- | --- | --- | --- | --- |
| 0 mM Mg^2+^ | 0.99 ± 0.03 | -19.51 ± 0.68 | 9.59 | -9.92 ± 0.68 | 261.4 ± 74.2 |
| 20 mM Mg^2+^ |  |  |  |  | ND |

**Cover Letter Table 1: Corrected ITC statistics.**

***“The Ni2+ toxicity experiment is nice, but really should have a few more control mutants. One would like to see that changing other positions in the central helix has no effect on function. If that could be demonstrated, it would make a stronger case for the importance of the N424 site.”***

We appreciate this constructive comment. Alanine-substituted mutations in the pore-lining residues typically induce the loss of MgtE transport activity (Hattori *et al*., EMBO J. 2009, PMID: 19798051). Only N332A and N424A mutants are known to maintain Mg^2+^‐auxotrophic *E. coli* growth complementation activity (Hattori *et al*., *EMBO J*. 2009, PMID: 19798051). Thus, in accordance with this comment, we will test the N332A mutant in the Ni^2+^ toxicity experiment.

***“**Minor comments:*****

***“The title is not accurate and should include mention of the Fab ex. 'Cryo-EM structure of a MgtE Mg^2+^ channel pore domain:Fab complex....'”***

In accordance with this comment, we will change the text in the revised manuscript.

***“Figure 1H (or any revised version thereof) needs a p value, or an indication that the differences are not significant.”***

We appreciate this comment, and we will add a p value (p = 0.62845) in Fig. 1H in the revised manuscript.

***“Fig. S1A and B could use labels to indicate low and high Mg2+, respectively.”***

In accordance with this comment, we will add labels in Fig. S1A and S1B in the revised manuscript. As we mentioned above, because we tested 0 mM MgCl_2_ instead of “low” Mg^2+^ concentrations in our experiments, we will add labels to indicate “Mg^2+^-free” and high-Mg^2+^ conditions.

***“Fig. S2 A needs labels for the peaks. (void, complex, etc.).”***

We appreciate this comment and will label the corresponding peaks as Reviewer #1 suggested.

***Reviewer #1 (Significance (Required)):***

***“The work provides new and potentially important insight into how MgtE functions and has the potential to fill in key gaps in our understanding of this protein and related channels.”***

We appreciate the positive response from Reviewer #1 regarding the significance of our work. We believe that the significance will be even more solid after addressing the Reviewer’s abovementioned concerns.

***Reviewer #2 (Evidence, reproducibility and clarity (Required)):***

***“If there was ever a transporter suffering from an "identity crisis" then MgtE would be the first in line. .... MgtE looks like an ABC transporter, is annotated as a secondary-active transporter, and functions as channel!***

***Previous models of MgtE have suffered from an inaccurate description of the proteins function. To muddy-the-waters, while the conduction rates of E. coli MgtE are consistent with an channel the human SLC homologue is reported to be a Na+/Mg2+ exchanger. Previous structures by Nureki's lab showed the access to the ion-binding site was blocked by plug helices in the presence of Mg2+, which acts in an auto-inhibitory manner by stabilising allosteric Mg2+ bound sites. However, key to understanding a translocation mechanism is the Mg2+ free structure of MgtE.***

***The authors in this paper report the cryo EM structure of Mg2+-free MgtE aided by a conformational-specific FAB fragment. They have validated the FAB complex by electrophysiology and cysteine cross-linking. This paper represents a major achievement. As anticipated, in the absence of Mg2+ the plug helices are disordered and the TM helices have moved apart at critical glycine resides. However, the structure is still blocked off towards the extracellular side, rather than the presence of an open Mg2+ channel. While the authors conclude this is a pre-open state, in my opinion, the structure looks like a transporter with a cavity in the inward-facing conformation. Indeed, the two-bundle movement when comparing Mg2+ free and Mg2+ present states look like transporter rearrangements, and not like the iris-like channel opening.***

***Overall, while I think this paper is a important breakthrough and technically sound for what is presented, I do not think the authors have been able to provide a mechanism that clarifies the function of MgtE ... it is still suffering from an identity crisis.”***

We appreciate the positive comment on our cryo-EM structure itself by stating that “this paper is an important breakthrough and technically sound for what is presented”, but we respectfully disagree with Reviewer #2’s view that it is not clear whether MgtE is an ion channel or a secondary active transporter. MgtE has been well established as an ion channel for over a decade. We would like to refute the points Reviewer #2 raised as potential reasons that MgtE may be a secondary active transporter.

First, we are confident that MgtE, in particular, *Thermus thermophiles* MgtE (TtMgtE), which is the protein used in this structural and functional characterization study, is an ion channel for the following reasons.

**1) High single channel conductance**

The single channel recording of TtMgtE exhibited 96±2.7 pS, corresponding to a transport efficiency of ∼3 × 10^7^ ions/s at −60 mV, which is much higher than those of active transporters (Hattori *et al*., *EMBO J*. 2009, PMID: 19798051).

**2) The channel activity of MgtE is not Na^+^-dependent.**

The single channel activity of TtMgtE was observed in the absence of any monovalent cations, such as Na^+^ and K^+^, by patch clamp recordings of both *E. coli* giant spheroplasts and proteoliposomes (Hattori *et al*., *EMBO J*. 2009, PMID: 19798051) (Tomita *et al.*, *Nature Communications*, 2017, PMID: 28747715). Furthermore, a proteoliposome-based assay using a Mg^2+^ fluorescent indicator showed that the Mg^2+^ transport activity was not affected by Na^+^ and K^+^. Thus, the Mg^2+^ transport of MgtE is unlikely to be driven by monovalent cations (Takeda *et al.*, *Nature Communications*, 2014, PMID: 25367295).

**3) The channel activity of MgtE is not proton-dependent.**

The single channel current of MgtE did not depend on the bath pH (Hattori *et al*., EMBO J. 2009, PMID: 19798051). Thus, the Mg^2+^ transport of MgtE is unlikely to be driven by protons.

**4) MgtE does not hydrolyse ATP**

Whereas the cytoplasmic domain of MgtE has ATP binding activity, no ATPase activity was detected in the ATP hydrolysis assay of MgtE. Furthermore, the single channel activity was still maintained upon “loss of ATP binding” mutations of MgtE (Tomita *et al.*, *Nature Communications*, 2017, PMID: 28747715).

Overall, these series of functional analyses strongly demonstrate that MgtE (in particular TtMgtE, which we have been employing for structural and functional analyses) is a channel instead of a secondary active transporter.

We next would like to refute the points that Reviewer #2 raised as potential reasons that MgtE may be a secondary active transporter.

***1) “MgtE is annotated as a secondary-active transporter”***

This is simply not the case. None of the bacterial MgtE proteins have been functionally annotated as a “secondary active transporter”. MgtE was simply “named” as a “transporter” when it was cloned based on genetics studies in 1995 (Smith, *et al*., J Bacteriol., 1995, PMID: 7868596) (Townsend, *et al*., J Bacteriol., 1995, PMID: 7665526). However, at that time, whether MgtE mediates passive transport (channel) or active transport (secondary active transporter) was not functionally characterized. Therefore, being named a “transporter” does not mean MgtE was annotated as a “secondary active transporter”. Many other membrane proteins that are named “transporters” also turned out to be channels after further functional characterization. The transport driving force of MgtE was not characterized until the single channel recording of TtMgtE in 2009, mentioned above (Hattori *et al*., EMBO J. 2009, PMID: 19798051).

As far as we know, only the Wikipedia website mentions MgtE as a potential secondary active transporter by stating that “*Kehres and Maguire suggest that the MgtE proteins are secondary carriers with inwardly directed polarity. [2]”* (<https://en.wikipedia.org/wiki/Magnesium_transporter_E>). No other research articles mention MgtE as a potential secondary active transporter.

This may cause a misunderstanding that MgtE might be a secondary active transporter. The paper cited by Wikipedia (Kehres, *et al*., Biometals, 2002, PMID: 12206392) also claimed the following:

*“MgtE sequences lack recognizable NTP binding motifs and therefore likely depend on the transmembrane electrochemical gradient to provide energy for Mg2+ transport.”* *“Energetics and molecular details of MgtE are completely unstudied.”*

Both channels and secondary active transporters are driven by electrochemical gradients (an electrical gradient and a chemical gradient), and it was clearly stated that the energetics of MgtE were unresolved. A similar description can be found in another previous review article (PMID: 16720382).

Therefore, the naming was confusing, but MgtE was not annotated as a “secondary active transporter”. To avoid such confusion, in the revised manuscript, we will add the abovementioned description on how bacterial MgtE, which was “named” as a “transporter”, has since been characterized as a channel.

***2) “MgtE looks like an ABC transporter”***

This is also simply not the case. MgtE is structurally and evolutionally totally distinct from an ABC transporter and is not driven by ATP hydrolysis. We surmise that Reviewer #2 meant to say that the conformation of MgtE looks like an inward-facing conformation of an ABC transporter (and other transporters). Such an idea has been a classical view before many membrane protein structures became available and does not support Reviewer #2’s view that MgtE may work as a secondary active transporter.

**Cover Letter Figure 1. Cross-sectional view of MgtE and other channels/transporters. (A**) The TM domain of MgtE in the presence (left panel) and absence (right panel) of Mg^2+^, **(B)** The TM domain of the ABC-type maltose transporter from *E. coli* (left panel: inward-facing, right panel: outward-facing), (**C**) Ctr1 copper transporter (named as a transporter, but works as a channel by mediating passive transport), (**D**) LRRC8A osmoregulated ion channel. The cartoon presentations are also shown.

As shown in **Cover Letter Figure 1**, in addition to the MgtE structures, other channels also adopt a partially open conformation with the cytoplasmic side solvent-accessible, similar to the cytoplasmic side of the ABC transporter, which is solvent-accessible in the inward-facing conformation. Thus, whether the cytoplasmic (or extracellular) side of the membrane protein structure is solvent-accessible does not correlate with whether it functions as a secondary active transporter.

***3) “The human SLC homologue is reported to be a Na+/Mg2+ exchanger.”***

It is true that the human SLC41 homologue, which is a eukaryotic counterpart of bacterial MgtE proteins, is reported to be a Na^+^/Mg^2+^ exchanger. However, it is not unusual that some family members function as a channel and other members function as a secondary active transporter, as in the case with CLC proteins. CLC family proteins consist of two distinct subgroups of Cl^−^ channels and Cl^−^/H^+^ transporters; nevertheless, they belong to the same CLC family. In particular, SLC41 proteins are quite distant from bacterial MgtE proteins. SLC41 possesses two tandemly repeated domains, which are homologues to the bacterial MgtE TM domain, whereas MgtE forms a dimer. Thus, it is not surprising that eukaryotic SLC41 functions as a secondary active transporter, whereas bacterial MgtE functions as a channel. To avoid confusion, we will add the corresponding descriptions regarding the difference between MgtE and SLC41 proteins in the revised manuscript.

*****Major comments.*****

***“I would suggest elastic modelling as an unbiased approach to establish if the intrinsic dynamics in MgtE resemble channel or transporter motions. MD simulations have been carried out here. Was it possible to measure Mg2+ transport in silico? Obviously, the reconstitution of MgtE into liposomes where function could be assessed in the presence and absence of Na+ gradient and a membrane potential would be very informative.”***

As we have already addressed Reviewer #2’s concern regarding whether MgtE is a channel or secondary active transporter, this has been addressed. The functional characterizations proposed by Reviewer #2 have been performed in the past, as we explained above.

*****Minor comments.*****

***“It was mentioned that a high concentration of magnesium ions can still close the MgtE channel by disrupting the MgtE-Fab complex. The FAB binds with high affinity. What do the grids of the MgtE-FB complex with high Mg2+ look like? Has the FAB fallen off?”***

We appreciate this comment. In contrast to the comment from Reviewer #1 saying that our Fab has relatively weak binding to MgtE, Reviewer #2 regards our Fab has having high affinity. We believe a *K*_d_ of ~250 nM indicates an intermediate case. Regarding the question raised, we have not checked grids of the MgtE-Fab in the presence of a high Mg^2+^ concentration. However, according to the FSEC-based binding assay (Figure S1C), the Fab fell off after adding the high concentration of Mg^2+^, and thus we would expect the same on the EM grids.

***“The authors mention that at 39kDa this represents one of the smallest structures determined by cryo EM. I would remove this statement as its a bit misleading since the protein is an oligomer (C2 symmetry was applied) so in actual fact that particles are 78kDa before the mass of the FAB is included. The total combined particle size is >100 kDa.”***

We apologize for our poor explanation that caused misunderstanding regarding the molecular weight of the MgtE TM domain dimer. It is indeed 39 kDa per dimer, not 78 kDa. The 39 kDa MgtE TM domain dimer is still the smallest among the membrane structure determinations by cryo-EM utilizing Fabs. Nevertheless, we fully respect the comment from Reviewer #2 and will delete the corresponding statement in the revised manuscript.

***“Other suggestions for improving the manuscript are listed below:***

***Abstract***

***Page 4, line 7-9***

***"However, due to the lack of a structure of the MgtE channel, including its transmembrane domain...has remained unclear"***

***This sentence needs to be rearranged as it reads as though there are not already structures solved. You could say something like: "There are no structures of the transmembrane domain for MgtE in Mg2+-free conditions, and the pore-opening mechanism has thus remained unclear."***

***Manuscript***

***General comment***

***Formatting of citations needs to be corrected throughout. Specifically, each citation is enclosed in a parenthesis where they can just be separated by a comma. E.g.: (Hartwig 2001)(Cown 2002)(Maguire and Cowan 2002)***

***Should read: (Hartwig 2001, Cown 2002, Maguire and Cowan 2002)***

***Page 5, lines 5-7.***

***"Therefore, homeostatic imbalance of Mg2+ ions causes multiple diseases, such as cardiovascular disease, high blood pressure, osteoporosis, and diabetes (Alexander et al. 2008)."***

***The statement that a homeostatic imbalance of Mg2+ ions causes multiple diseases, is overstated and inaccurate. For example, in Alexander et al. 2008 it states "current data, although by no means conclusive, suggests that Mg2+ deficiency predisposes an individual to osteoporosis. The reference also makes no comment regarding how a homeostatic imbalance of Mg2+ ions causes diabetes or high blood pressure.***

***Page 5, line 10***

***"MgtE belongs to a family of Mg2+ channels that is ubiquitously distributed "***

***Should read: MgtE belongs to a family of Mg2+ channels that are ubiquitously distributed***

***Page 6, line 2***

***Specify the type of diabetes, i.e., type 2***

***Page 6, line 15-16***

***"where the N domain is dissociated from the CBS domain and where each subunit of the CBS domain and plug helix dimer are also separated from each other"***

***When comparing these differences in the structures, approximately how far have they moved?***

***Page 8-9, line 16 (page 8) to line 3 (page 9)***

***"We then performed patch clamp analysis of full-length MgtE using Escherichia coli giant spheroplasts at 0 mM MgCl2 in the presence and absence of Fab705 and observed little change in the open probabilities upon addition of Fab705 into the bath solution (Fig. 1F and 1H). "***

***Comment: There is no reference to Figure 1G. Instead it is mentioned on Page 12, line 7.***

***"Consistent with this, in the patch clamp recording, when we tested a high concentration of MgCl2 (20 mM MgCl2) in the bath solution after the addition of Fab705, we did not observe channel opening, as similarly observed with a high concentration of MgCl2 in the absence of Fab705 (Fig. 1G)."***

***I think the comment on page 12 lines 4-7 should be combined with the above.***

***Page 8, line 3***

***"These results showed"***

***Comment: Be careful with tenses. It should read show, or demonstrate. I suggest: "These results demonstrate"***

***Page 8, line 4***

***"MgtE at high" should read "MgtE with high"***

***Page 8, line 6***

***"functional properties of Fab705 would be desirable". Should read "functional properties of Fab705 are desirable"***

***Page 8, lines 8-9***

***"Thus, the utilization of Fab705 would enable us to capture the gating motions of MgtE by cryo-EM." Should read "Thus, the utilization of Fab705 enables the gating motions of MgtE to be captured by cryo-EM."***

***Page 8, lines 11-13***

***I suggest rewriting this sentence: "We performed cryo-EM single-particle analysis to determine the structure of the full-length MgtE-Fab705 complex reconstituted in PMAL-C8 amphipol in the presence of 1 mM EDTA and the absence of divalent cations (Fig. 2, S2 and S3)" to "To determine the structure of MgtE in the absence of Mg2+ or other divalent cations, the full-length MgtE-Fab705 complex was reconstituted in PMAL-C8 amphipol and subjected to cryo-EM single-particle analysis (Fig. 2, S2 and S3).***

***Details such as "in the presence of 1 mM EDTA" should be in the methods with the other buffer conditions.***

***Page 8, line 14***

***"images was performed" should read "images were performed"***

***Page 10, line 1***

***"domain was clear enough to build an atomic model"***

***Comment: Can you add the resolution of this domain?***

***Page 10-11, line 16 (page10) to line 2 (page 11)***

***"and we successfully visualized the conformation of the TM domain under Mg2+-free conditions for the first time, to the best of our knowledge."***

***I suggest re-writing this sentence to read: "and for the first time we have successfully visualized the conformation of the TM domain under Mg2+-free conditions."***

***Page 12, line 11***

***You should state the PDB ID's throughout when referring to previously solved structures.***

***Page 12, line 14***

***What structure are you referring to? If it is the closed one with Mg2+then state that.***

***Page 12, line 15***

***"showed" should be "shows" or you could use "has"***

***Page 13, second paragraph***

***Can you comment on why this was not tested with Mg2+? Was it attempted and it did not work? Or is it not plausible to do such an experiment? Please add these details to the manuscript.***

***Page 13, lines 8-11***

***"We conducted a Ni2+ sensitivity assay of E. coli with the N424A mutant of MgtE; Mg2+ channels, including MgtE, typically transport Ni2+ because the coordination chemistry of these ions is highly similar to that of Mg2+ (Moomaw and Maguire 2008)."***

***This sentence needs to be re-written. I suggest the following: "We conducted a Ni2+ sensitivity assay in E. coli using the N424A-MgtE mutant. Because the the coordination chemistry required for Ni2+ is highly similar to that of Mg2+, MgtE and other Mg2+ channels typically transport Ni2+ (Moomaw and Maguire 2008)."***

***Page 13, last paragraph***

***"E. coli cells expressing the N424A mutant of MgtE exhibited increased Ni2+ sensitivity (Fig. 4D) compared to cells expressing wild-type MgtE, suggesting that disruption of the cytoplasmic gate led to increased Ni2+ uptake."***

***This should be moved up and joined with the paragraph above, rather than standing alone.***

***Page 14, lines 13-15***

***Furthermore, the TM1 helices that are directly connected to the plug helices have also moved away from the center, as they interact with both the TM2 and TM5 helices (Fig. 5B).***

***Can you add how far these have moved?***

***Page 15, lines 4-6***

***"Using these mutants with Cu2+ phenanthroline as a catalyst, we performed cross-linking experiments (Fig. 5D and 5E)."***

***I suggest re-writing this to "Using Cu2+ phenanthroline as a catalyst, cross-linking experiments were performed with these mutants (Fig. 5D and 5E)."***

***Page 17, lines 7-9***

***"While the expression of wild-type MgtE rescued Mg2+-auxotrophic growth, the mutations at these glycine residues almost abolished Mg2+ transport activity (Fig. 6C)."***

***Comment: Where is the data showing transport activity? If this was not performed, then it should just say growth complementation. Can you show data for bacterial growth?***

***Page 18, lines 7-10***

***"To be noted, the functional properties of Fab705 would be desirable for determination of the MgtE structure under Mg2+-free conditions because it does not affect the channel gating under Mg2+-free conditions, in particular in an inhibitory manner (Fig. S8)."***

***This sentence does not make sense. Please re-write in a clearer way.***

***Page 18, second to last paragraph.***

***"Based on this structure, we further conducted structure-based functional analysis, and our new findings together with our previous research on MgtE provided structural insights into the gating mechanism of MgtE, as discussed below."***

***This should be joined with the sentence paragraph instead of standing alone.***

***Page 19, lines 3-4***

***"In contrast, under Mg2+-free conditions, the MgtE cytoplasmic domain, including the plug helices, was totally disordered in our cryo-EM map (Fig. 2)"***

***Comment: This is not what Fig.2 shows. Instead you could say that these domains could not be built into the cryo-EM map.***

***“Page 20, line 7 "1B and 7B)."***

***Comment: The "B)" should be in bold.”***

***Figure legends and figures***

***General comment***

***Helices in legends are labelled as red, but this is not red. You could use salmon.***

***Figure 1 Legend The "(A)" should be in bold. To the figure you could add arrows to show movement of the N-domain (B) and plug domain (C). Maybe even the distance it has moved could be added to the figure. In (B) and (C) please add more details to the legend, such as which colour refers to what. For (F) and (G) please add more details, such as what the 90 inside the cone means.***

***Figure 1D and 1E In the methods it states that the ITC experiments were repeated three times with similar results. There are however no details of this in the figure legend. Is the data you are showing an average of these experiments? Or representative? Please include these details in the figure legend. I suggest showing all of the data points for each run, or the error.***

***Figure 2 Legend Add "Representative cryo-EM image" to "Cryo-EM image". Be more specific for (D-F) with what each is showing. E.g. (D) side views, (E) top-views etc.***

***Figure 2 Label the domains and views.***

***Figure 3 Label the views.***

***Figure 4 Legend Add PDB IDs where required***

***Figure 4 You could highlight the residues you mention in the text a different colour. This figure is also showing different structures, then I would use different colour ways for the different structures, and also label them accordingly.***

***Figure 5 Legend Add PDB IDs where required. In part D, you need more detail regarding what the figure is showing. E.g. gel. You also say the experiments were repeated six times. Can you show the other gels in supplementary? Is the error in the graph the SEM? If so, state it in the legend.***

***Figure 7 Legend Add "A cartoon representation of the proposed" to "A cartoon of the proposed" Add "The yellow circles containing the letter G" to "The yellow circles containing G"***

***Supplementary***

***Figure S1 Label graphs with conditions, i.e. Mg2+-free, 20 mM Mg2+. It is easier for the reader this way.***

***Can you comment on why the MgtE in magnesium free conditions (orange) is further to the left? This peak elutes in the same position as part A of the figure, with or without Fab so it seems it is to do with the absence of Mg2+?***

***Page 1, lines 6-9 Part C is not clear. It is helpful for the reader if you refer to the color on the graph in the text. This would be helpful to do throughout the manuscript.***

***Figure S2 I think it is also helpful here to label the figure with void, Fab-MgtE free-Fab. You could also highlight which area was pooled for cryo-EM data collection.***

***Figure S3 (A) And (B) letters need to be in bold so it is consistent.***

***I also suggest labelling the TMs and the first and last residues in the figure, rather than just the legend.***

***Figure S4 Bold the letters (A), (B) etc.***

***Figure S5 Lacking details in the legend such as colours.***

***Figure S6 Bold the letter (A)***

***Table S1 I don't think you need to show a row for "With Mg2+ as there is no data, and it has been mentioned in the text.”***

We appreciate these comments regarding the textual changes and the figure presentation and formatting. We will make changes based on these comments in the revised manuscript.

***Reviewer #2 (Significance (Required)):***

***“The MgtE family is mechanistically an interesting member as it "blurs" the line between a channel and a transporter. The structure of Mg2+ free MgtE is an important achievement and this structure, in of its own right, will push the field forward. However, if the authors were to invest more time ca. 3 months in developing their model with the current structure (e.g. MD simulations showing Mg2+ conductance, dynamics) it could clarify if and how MgtE functions as a channel? If it doesnt, then perhaps the door should still be left open that this family might be a transporter ...”***

We appreciate the positive comments on the significance of our cryo-EM study (*The structure of Mg^2+^-free MgtE is an important achievement, and this structure, on its own right, will push the field forward*.). However, we respectfully disagree with Reviewer 3’s view that MgtE may be a secondary active transporter. The misunderstanding that MgtE may be a secondary active transporter has been sufficiently addressed above. Furthermore, according to the request of Reviewer #2, we will run the MD simulations over a longer period to see if we can observe some structural changes.

***Reviewer #3 (Evidence, reproducibility and clarity (Required)):***

***“The study reported by Jin et al. describes the structure and functional analysis of the bacterial magnesium channel MgtE. The authors address an important and outstanding question in the field, which relates to the role of intracellular magneduium in regulating the open/closed state of the channel. The current work reports the structure of MgtE in the absence of Mg2+ ions, revealing a plausible mechanism for regulation. The authors propose a model whereby Mg2+ binding to the cytoplasmic regulatory domain closes the channel by ordering these domains enabling a plug domain to block the pore. The current structure reveals that following Mg2+ dissasocition teh regulatory domain adopts a flexible structure that enables the plug to release and channel to adopt a conducting state.”***

We appreciate the positive comments from Reviewer #3.

***Reviewer #3 (Significance (Required)):***

***“The work is significant for revealing an important state of the MgtE channel and for proposing a plausible model for Mg2+ depedent activation. The paper is well written and the results presented clearly and logically. It would be nice to add some references to similar types of regulation observed in other channels with cytoplasmic regulatory domains, where I understand similar order-disorder transitions have been proposed as regulatory models. The use of FABs is gaining increased traction for determining the structures of small membrane proteins, and I think this study also serves as a good example in this context.”***

We appreciate the positive comments from Reviewer #3. In accordance with this comment, in the revised manuscript, we will add references regarding similar types of regulation observed in other channels cytoplasmic regulatory domains.
