# Supplementary material for: The structure of MgtE in the absence of magnesium provides new insights into channel gating
Source: PLoS Biol. 2021 Apr 27;19(4):e3001231. doi: 10.1371/journal.pbio.3001231 (PMC8104411; doi:10.1371/journal.pbio.3001231)
Supplement: Attachment — Submitted filename: Response-to-Reviewers-MgtE-PloSBio-Rev1.docx [file pbio.3001231.s022.docx]

**Reviewer #1 (Evidence, reproducibility and clarity (Required)):**

***“The manuscript by Jin and coworkers reports the cryo-EM structure of the Thermus thermophilus MgtE magnesium ion channel in Mg2+ free conditions bound to an antibody Fab, Fab705. The structure shows a complex in which each subunit of the channel dimer is bound by one Fab705. The conformation shows an open intracellular side, but also shows that the channel remains closed on the extracellular side. The cytoplasmic assembly that is stabilized by Mg2+ is invisible and it is inferred that it has become mobile. Further, the binding of Fab705 to the intracellular side of the transmembrane domains is clearly structurally incompatible with the positions of the intracellular domains in the closed channel state. The authors use crosslinking and functional studies to validate key interactions and conformational changes inferred from this structure relative to the closed MtgE. Overall, the data are mostly convincing but there are a few issues that must be addressed.”***

We appreciate the positive response from Reviewer #1. We addressed the specific comments below.

***“**Major comments:*****

***“The stoichiometry for the ITC is substantially different from the expected 1:1. This is likely due to errors in determining the concentration of the components. It is not stated in the methods how these were measured and the use of symbols indicting approximate values indicates that these experiments were not done with the accuracy required for meaningful analysis.”***

We thank the reviewer for this comment. The protein concentration was estimated using absorbance at 280 nm. After checking the experimental records, we realized our simple mistake in the Fab concentration value we used in analyzing the ITC data. The calculated absorbance values at 280 nm for 1 mg/ml are 2.071 and 1.42 for the Fab heavy and light chains, respectively, but we mistakenly applied 1.0 for both when we analyzed the ITC data. After correcting this, the stoichiometry was closer to 1:1 (now N=0.89 ± 0.02, previously N=1.32 ± 0.03), and the *K*_d_ value of 216.1 ± 52.7 (nM) was still comparable to the previous *K*_d_ value (252.6 ± 71.1 nM) (**Table S1**). Furthermore, according to the request from Reviewer #2, we included all ITC results we performed three times by adding the data for the other two trials (**Fig. S10 and Table S1**). Notably, we did not find this type of mistake in any other experiment. We apologize for our carelessness, and we corrected the associated figure, table and descriptions based on the corrected ITC analysis results in the revised manuscript (**Fig. S10 and Table S1**) (From Page 8, Line 14 to Page 9, Line 2) (Page 30, Lines 8-10).

***“The Ni2+ toxicity experiment is nice, but really should have a few more control mutants. One would like to see that changing other positions in the central helix has no effect on function. If that could be demonstrated, it would make a stronger case for the importance of the N424 site.”***

We thank the reviewer for this constructive comment. Alanine-substituted mutations in the pore-lining residues typically induce the loss of MgtE transport activity (Hattori *et al*., EMBO J. 2009, PMID: 19798051); thus, most mutations of the pore-lining residues are not suitable for the Ni^2+^ toxicity experiment. Only the N332A and N424A mutants are known to maintain Mg^2+^‐auxotrophic *E. coli* growth complementation activity (Hattori *et al*., *EMBO J*. 2009, PMID: 19798051). Thus, in accordance with this comment, in addition to the N424A mutant, we newly tested the N332A mutant in the Ni^2+^ toxicity experiment and found that both the N332A and N424A mutants of MgtE exhibited increased Ni^2+^ sensitivity (**Fig. 4D**) (Page 13, Line 12 to Page 14, Line 10). This could be because N332 also contributes to the formation of the cytoplasmic gate by closely contacting N424. To show the close contacts of N332 with N424, including hydrogen bonding, we made a new figure to show a close-up view of the cytoplasmic gate (**Fig. 4B**).

***“**Minor comments:*****

***“The title is not accurate and should include mention of the Fab ex. 'Cryo-EM structure of a MgtE Mg^2+^ channel pore domain:Fab complex....'”***

In accordance with this comment, we changed the title in the revised manuscript (Page 1).

***“Figure 1H (or any revised version thereof) needs a p value, or an indication that the differences are not significant.”***

We appreciate this comment, and we added a p value (p = 0.804) to the figure legend of Fig. 1H in the revised manuscript.

***“Fig. S1A and B could use labels to indicate low and high Mg2+, respectively.”***

In accordance with this comment, we added labels in Fig. S1A and S1B in the revised manuscript. As we tested 0 mM MgCl_2_ in our experiments, we added labels to indicate “Mg^2+^-free” and “high-Mg^2+^” conditions (**Fig. S1**).

***“Fig. S2 A needs labels for the peaks. (void, complex, etc.).”***

We appreciate this comment and labeled the corresponding peaks as Reviewer #1 suggested (**Fig. S2A**).

***Reviewer #1 (Significance (Required)):***

***“The work provides new and potentially important insight into how MgtE functions and has the potential to fill in key gaps in our understanding of this protein and related channels.”***

We appreciate the positive response from Reviewer #1 regarding the significance of our work. We believe that the significance would be even better after addressing the Reviewer’s abovementioned concerns.

***Reviewer #2 (Evidence, reproducibility and clarity (Required)):***

***“If there was ever a transporter suffering from an "identity crisis" then MgtE would be the first in line. .... MgtE looks like an ABC transporter, is annotated as a secondary-active transporter, and functions as channel!***

***Previous models of MgtE have suffered from an inaccurate description of the proteins function. To muddy-the-waters, while the conduction rates of E. coli MgtE are consistent with an channel the human SLC homologue is reported to be a Na+/Mg2+ exchanger. Previous structures by Nureki's lab showed the access to the ion-binding site was blocked by plug helices in the presence of Mg2+, which acts in an auto-inhibitory manner by stabilising allosteric Mg2+ bound sites. However, key to understanding a translocation mechanism is the Mg2+ free structure of MgtE.***

***The authors in this paper report the cryo EM structure of Mg2+-free MgtE aided by a conformational-specific FAB fragment. They have validated the FAB complex by electrophysiology and cysteine cross-linking. This paper represents a major achievement. As anticipated, in the absence of Mg2+ the plug helices are disordered and the TM helices have moved apart at critical glycine resides. However, the structure is still blocked off towards the extracellular side, rather than the presence of an open Mg2+ channel. While the authors conclude this is a pre-open state, in my opinion, the structure looks like a transporter with a cavity in the inward-facing conformation. Indeed, the two-bundle movement when comparing Mg2+ free and Mg2+ present states look like transporter rearrangements, and not like the iris-like channel opening.***

***Overall, while I think this paper is a important breakthrough and technically sound for what is presented, I do not think the authors have been able to provide a mechanism that clarifies the function of MgtE ... it is still suffering from an identity crisis.”***

We appreciate the positive comment on our cryo-EM structure itself by stating that “*this paper is an important breakthrough and technically sound for what is presented*”, but we respectfully disagree with Reviewer #2’s view that it is not clear whether MgtE is an ion channel or a secondary active transporter. MgtE has been well established as an ion channel for over a decade. We would like to refute the points Reviewer #2 raised as potential reasons that MgtE may be a secondary active transporter.

First, we are confident that MgtE, in particular, *Thermus thermophiles* MgtE (TtMgtE), which is the protein used in this structural and functional characterization study, is an ion channel for the following reasons.

**1) High single channel conductance**

The single channel recording of TtMgtE exhibited 96±2.7 pS, corresponding to a transport efficiency of ∼3 × 10^7^ ions/s at −60 mV, which is much higher than that of active transporters (Hattori *et al*., *EMBO J*. 2009, PMID: 19798051).

**2) The channel activity of MgtE is not Na^+^-dependent.**

The single channel activity of TtMgtE was observed in the absence of any monovalent cations, such as Na^+^ and K^+^, by patch clamp recordings of both *E. coli* giant spheroplasts and proteoliposomes (Hattori *et al*., *EMBO J*. 2009, PMID: 19798051) (Tomita *et al.*, *Nature Communications*, 2017, PMID: 28747715). Furthermore, a proteoliposome-based assay using a Mg^2+^ fluorescent indicator showed that Mg^2+^ transport activity was not affected by Na^+^ and K^+^. Thus, the Mg^2+^ transport of MgtE is unlikely to be driven by monovalent cations (Takeda *et al.*, *Nature Communications*, 2014, PMID: 25367295).

**3) The channel activity of MgtE is not proton-dependent.**

The single channel current of MgtE does not depend on the bath pH (Hattori *et al*., EMBO J. 2009, PMID: 19798051). Thus, the Mg^2+^ transport of MgtE is unlikely to be driven by protons.

**4) MgtE does not hydrolyze ATP**

Whereas the cytoplasmic domain of MgtE has ATP binding activity, no ATPase activity was detected in the ATP hydrolysis assay of MgtE. Furthermore, single channel activity was still maintained upon “loss of ATP binding” mutations of MgtE (Tomita *et al.*, *Nature Communications*, 2017, PMID: 28747715).

Overall, this series of functional analyses strongly demonstrates that MgtE (in particular TtMgtE, which we employed for structural and functional analyses) is a channel instead of a secondary active transporter. We added a description of the previous patch clamp recording of MgtE in the introduction section (Page 5, Lines 10-15).

We next would like to refute the points that Reviewer #2 raised as potential reasons that MgtE may be a secondary active transporter.

***1) “MgtE is annotated as a secondary-active transporter”***

I respectfully disagree to this view. None of the bacterial MgtE proteins have been functionally annotated as a “secondary active transporter”. MgtE was simply “named” as a “transporter” when it was cloned based on genetic studies in 1995 (Smith, *et al*., J Bacteriol., 1995, PMID: 7868596) (Townsend, *et al*., J Bacteriol., 1995, PMID: 7665526). However, at that time, whether MgtE mediates passive transport (channel) or active transport (secondary active transporter) was not functionally characterized. Therefore, being named a “transporter” does not mean MgtE was annotated as a “secondary active transporter”. Many other membrane proteins that have been named “transporters” also turned out to be channels after further functional characterization. The transport driving force of MgtE was not characterized until the single channel recording of TtMgtE in 2009, as mentioned above (Hattori *et al*., EMBO J. 2009, PMID: 19798051).

To the best of our knowledge, only the Wikipedia website mentions MgtE as a potential secondary active transporter by stating that “*Kehres and Maguire suggest that the MgtE proteins are secondary carriers with inwardly directed polarity. [2]”* (<https://en.wikipedia.org/wiki/Magnesium_transporter_E>).

This may cause a misunderstanding that MgtE might be a secondary active transporter. However, the paper cited by Wikipedia (Kehres, *et al*., Biometals, 2002, PMID: 12206392) also claimed the following:

*“MgtE sequences lack recognizable NTP binding motifs and therefore likely depend on the transmembrane electrochemical gradient to provide energy for Mg2+ transport.”* *“Energetics and molecular details of MgtE are completely unstudied.”*

Both channels and secondary active transporters are driven by electrochemical gradients (an electrical gradient and a chemical gradient), and it was clearly stated that the energetics of MgtE were unresolved.

Therefore, the naming was slightly confusing, but MgtE was not annotated as a “secondary active transporter”. To avoid such confusion, in the revised manuscript, we added the abovementioned description on how bacterial MgtE, which was originally “named” a “transporter”, has since been characterized and is a channel (Page 5, Lines 10-15).

***2) “MgtE looks like an ABC transporter”***

MgtE is structurally and evolutionally distinct from an ABC transporter and is not driven by ATP hydrolysis. We surmise that Reviewer #2 meant to say that the conformation of MgtE looks like an inward-facing conformation that is similar to that of an ABC transporter (and other transporters). However, such an idea has been a classical view before many membrane protein structures became available and does not support Reviewer #2’s view that MgtE may work as a secondary active transporter.

**Cover Letter Figure 1. Cross-sectional view of MgtE and other channels/transporters. (A**) The TM domain of MgtE in the presence (left panel) and absence (right panel) of Mg^2+^. **(B)** The TM domain of the ABC-type maltose transporter from *E. coli* (left panel: inward-facing, right panel: outward-facing). (**C**) Ctr1, a copper transporter (named as a transporter but works as a channel by mediating passive transport). (**D**) LRRC8A, an osmoregulation ion channel. The cartoon presentations are also shown.

As shown in **Cover Letter Figure 1**, in addition to the MgtE structures, other channels also adopt a partially open conformation with the cytoplasmic side solvent accessible, similar to the cytoplasmic side of the ABC transporter, which is solvent accessible in the inward-facing conformation. Thus, whether the cytoplasmic (or extracellular) side of the membrane protein structure is solvent accessible does not correlate with whether it functions as a secondary active transporter. Likewise, there are multiple ion channels that do not open in an iris-like manner such as CLC, TMEM16A, CFTR, and Hv1 channels. Thus, whether MgtE opens in an iris-like manner also does not correlate with whether it functions as a channel or a secondary active transporter.

***3) “The human SLC homologue is reported to be a Na+/Mg2+ exchanger.”***

It is true that the human SLC41 homologue, which is a eukaryotic counterpart of bacterial MgtE proteins, is reported to be a Na^+^/Mg^2+^ exchanger. However, it is not unusual that some family members function as a channel and other members function as a secondary active transporter, as in the case of CLC proteins. CLC family proteins consist of two distinct subgroups of Cl^−^ channels and Cl^−^/H^+^ transporters; nevertheless, they belong to the same CLC family. In particular, SLC41 proteins are quite distant from bacterial MgtE proteins. SLC41 possesses two tandemly repeated domains, which are homologs to the bacterial MgtE TM domain, whereas MgtE forms a dimer. Thus, it is not surprising that eukaryotic SLC41 functions as a secondary active transporter, whereas bacterial MgtE functions as a channel. To avoid confusion, we added the corresponding descriptions of SLC41 proteins as secondary active transporters and cited papers on the CLC family proteins in the introduction section of the revised manuscript (Page 6, Lines 3-6).

*****Major comments.*****

***“I would suggest elastic modelling as an unbiased approach to establish if the intrinsic dynamics in MgtE resemble channel or transporter motions. MD simulations have been carried out here. Was it possible to measure Mg2+ transport in silico? Obviously, the reconstitution of MgtE into liposomes where function could be assessed in the presence and absence of Na+ gradient and a membrane potential would be very informative.”***

As we have already addressed Reviewer #2’s concern regarding whether MgtE is a channel or secondary active transporter; this has been addressed above. Functional characterizations to determine whether MgtE is an ion channel or a secondary transporter have been performed by patch clamp recordings of both *E. coli* giant spheroplasts and proteoliposomes as well as a proteoliposome-based assay using a Mg^2+^ fluorescent indicator in the past, as we explained above. If MgtE is a secondary active transporter, single channel currents cannot be recorded, particularly from liposomes with a purified protein. Single channel recording with purified proteins has been employed to provide direct evidence for ion channels, for instance, the case of RyR (Lai *et al*., Nature 1988, PMID: 2448641) and CFTR (Bear *et al*., Cell 1992, PMID: 1371239). Thus, MgtE also has been shown to be an ion channel. Furthermore, the MD simulation would not be a suitable method to determine whether MgtE is an ion channel or a secondary active transporter. Only functional characterizations can solely determine it. Nevertheless, to fully respect the comment from Reviewer #2, we spent three months to run new MD simulations over a longer period of time (**Fig. S8**). Please see the response below.

*****Minor comments.*****

***“It was mentioned that a high concentration of magnesium ions can still close the MgtE channel by disrupting the MgtE-Fab complex. The FAB binds with high affinity. What do the grids of the MgtE-FB complex with high Mg2+ look like? Has the FAB fallen off?”***

We thank the reviewer for this comment. Regarding the question raised, we did not check the grids of MgtE-Fab in the presence of a high Mg^2+^ concentration. However, according to the result of the FSEC-based binding assay (**Fig. S1C**), the Fab fell off after adding a high concentration of Mg^2+^; thus, we would expect the same on the EM grids.

***“The authors mention that at 39kDa this represents one of the smallest structures determined by cryo EM. I would remove this statement as its a bit misleading since the protein is an oligomer (C2 symmetry was applied) so in actual fact that particles are 78kDa before the mass of the FAB is included. The total combined particle size is >100 kDa.”***

We apologize for our poor explanation, which caused a misunderstanding regarding the molecular weight of the MgtE TM domain dimer. The MgtE TM domain dimer is indeed 39 kDa per dimer, not 78 kDa. The 39 kDa MgtE TM domain dimer is still the smallest among the membrane structure determinations by cryo-EM utilizing Fabs. Nevertheless, we fully respected the comment from Reviewer #2 and removed the corresponding statement in the revised manuscript (Page 18).

***“Other suggestions for improving the manuscript are listed below:***

***Abstract***

***Page 4, line 7-9***

***"However, due to the lack of a structure of the MgtE channel, including its transmembrane domain...has remained unclear"***

***This sentence needs to be rearranged as it reads as though there are not already structures solved. You could say something like: "There are no structures of the transmembrane domain for MgtE in Mg2+-free conditions, and the pore-opening mechanism has thus remained unclear."***

We corrected the corresponding sentence (Page 4, Lines 7-8).

***Manuscript***

***General comment***

***Formatting of citations needs to be corrected throughout. Specifically, each citation is enclosed in a parenthesis where they can just be separated by a comma. E.g.: (Hartwig 2001)(Cown 2002)(Maguire and Cowan 2002)***

***Should read: (Hartwig 2001, Cown 2002, Maguire and Cowan 2002)***

We apologize for our carelessness. We corrected our citation formatting to follow the suggestion from Reviewer #2 and the citation format of PLOS Biology.

***Page 5, lines 5-7.***

***"Therefore, homeostatic imbalance of Mg2+ ions causes multiple diseases, such as cardiovascular disease, high blood pressure, osteoporosis, and diabetes (Alexander et al. 2008)."***

***The statement that a homeostatic imbalance of Mg2+ ions causes multiple diseases, is overstated and inaccurate. For example, in Alexander et al. 2008 it states "current data, although by no means conclusive, suggests that Mg2+ deficiency predisposes an individual to osteoporosis. The reference also makes no comment regarding how a homeostatic imbalance of Mg2+ ions causes diabetes or high blood pressure.***

We apologize for the overstatement. We removed the corresponding sentence from the revised manuscript (Page 5).

***Page 5, line 10***

***"MgtE belongs to a family of Mg2+ channels that is ubiquitously distributed "***

***Should read: MgtE belongs to a family of Mg2+ channels that are ubiquitously distributed***

We thank the reviewer for this comment. We have corrected the corresponding sentence (Page 5, Lines 7-8).

***Page 6, line 2***

***Specify the type of diabetes, i.e., type 2***

We thank the reviewer for this comment and corrected the corresponding sentence (Page 6, Line 2).

***Page 6, line 15-16***

***"where the N domain is dissociated from the CBS domain and where each subunit of the CBS domain and plug helix dimer are also separated from each other"***

***When comparing these differences in the structures, approximately how far have they moved?***

We appreciate this comment from Reviewer #2. In Fig. 1B and 1C of the revised manuscript, we added black arrows and red bars, denoting the rotation of the N domain and plug helices, and referred to Fig. 1B and 1C in the corresponding sentence (**Fig. 1B and 1C**) (Page 7, Line 4).

***Page 8-9, line 16 (page 8) to line 3 (page 9)***

***"We then performed patch clamp analysis of full-length MgtE using Escherichia coli giant spheroplasts at 0 mM MgCl2 in the presence and absence of Fab705 and observed little change in the open probabilities upon addition of Fab705 into the bath solution (Fig. 1F and 1H). "***

***Comment: There is no reference to Figure 1G. Instead it is mentioned on Page 12, line 7.***

***"Consistent with this, in the patch clamp recording, when we tested a high concentration of MgCl2 (20 mM MgCl2) in the bath solution after the addition of Fab705, we did not observe channel opening, as similarly observed with a high concentration of MgCl2 in the absence of Fab705 (Fig. 1G)."***

***I think the comment on page 12 lines 4-7 should be combined with the above.***

According to this comment, we combined the corresponding sentences (Page 9, Lines 5-8).

***Page 8, line 3***

***"These results showed"***

***Comment: Be careful with tenses. It should read show, or demonstrate. I suggest: "These results demonstrate"***

We corrected the corresponding sentence (Page 9, Line 8).

***Page 8, line 4***

***"MgtE at high" should read "MgtE with high"***

We appreciate this comment and corrected the corresponding sentence (Page 9, Line 9).

***Page 8, line 6***

***"functional properties of Fab705 would be desirable". Should read "functional properties of Fab705 are desirable"***

By following the suggestion from Reviewer #2, we corrected the corresponding sentence (Page 9, Line 11).

***Page 8, lines 8-9***

***"Thus, the utilization of Fab705 would enable us to capture the gating motions of MgtE by cryo-EM." Should read "Thus, the utilization of Fab705 enables the gating motions of MgtE to be captured by cryo-EM."***

We appreciate the comment and corrected the corresponding sentence (Page 9, Line14).

***P****ag****e 8, lines 11-13***

***I suggest rewriting this sentence: "We performed cryo-EM single-particle analysis to determine the structure of the full-length MgtE-Fab705 complex reconstituted in PMAL-C8 amphipol in the presence of 1 mM EDTA and the absence of divalent cations (Fig. 2, S2 and S3)" to "To determine the structure of MgtE in the absence of Mg2+ or other divalent cations, the full-length MgtE-Fab705 complex was reconstituted in PMAL-C8 amphipol and subjected to cryo-EM single-particle analysis (Fig. 2, S2 and S3).***

***Details such as "in the presence of 1 mM EDTA" should be in the methods with the other buffer conditions.***

By following the suggestion from Reviewer #2, we corrected the corresponding sentence (Page 10, Lines 1-3).

***Page 8, line 14***

***"images was performed" should read "images were performed"***

We appreciate this comment, but the subject of this sentence is “A 3D reconstruction”, not “images”. Thus, I think we do not need to correct this sentence. In addition, we had native English speakers proofread our revised manuscript, and this sentence is grammatically correct.

***Page 10, line 1***

***"domain was clear enough to build an atomic model"***

***Comment: Can you add the resolution of this domain?***

To follow this comment, we prepared an additional cryo-EM density map of the TM domain that was calculated at an ~3.1 Å resolution by signal subtraction and additional 3D refinement (**new Fig. S5**) (Page 10, Lines 6-8).

***Page 10-11, line 16 (page10) to line 2 (page 11)***

***"and we successfully visualized the conformation of the TM domain under Mg2+-free conditions for the first time, to the best of our knowledge."***

***I suggest re-writing this sentence to read: "and for the first time we have successfully visualized the conformation of the TM domain under Mg2+-free conditions."***

We appreciate the comment and corrected the corresponding sentence (Page 11, Lines 6-8).

***Page 12, line 11***

***You should state the PDB ID's throughout when referring to previously solved structures.***

We added the PDB IDs in the sentence Reviewer #2 pointed out as well as throughout the figure legends so that readers know which structures are being referring to (Pages 12, Lines 51-16) (Legend of Fig. 1, 4, 5, 6, S6 and S7).

***Page 12, line 14***

***What structure are you referring to? If it is the closed one with Mg2+then state that.***

We appreciate the comment and corrected the corresponding sentence (Page 13, Lines 4-5).

***Page 12, line 15***

***"showed" should be "shows" or you could use "has"***

By following the suggestion from Reviewer #2, we corrected the corresponding sentence (Page 13, Line 5).

***Page 13, second paragraph***

***Can you comment on why this was not tested with Mg2+? Was it attempted and it did not work? Or is it not plausible to do such an experiment? Please add these details to the manuscript.***

We thank the reviewer for this comment. We did not test these mutants with Mg^2+^ because Mg^2+^ is much less toxic than Ni^2+^, so it is virtually impossible to perform such a sensitivity assay with Mg^2+^ in *E. coli*. Instead, heavy atoms, with a coordination chemistry similar to that of Mg^2+^_,_ such as Ni^2+^ and Co^2+^, have been employed for such sensitivity assays in *E. coli*, as they are toxic. The change in the sensitivities to Ni^2+^ was already employed to characterize MgtE and its mutants in a previous MgtE paper (Tomita *et al*., Nature Communications, 2017, PMID: 28747715). We added the corresponding description in the revised manuscript (From Page 15, Line 13 to Page 14, Line 7).

***Page 13, lines 8-11***

***"We conducted a Ni2+ sensitivity assay of E. coli with the N424A mutant of MgtE; Mg2+ channels, including MgtE, typically transport Ni2+ because the coordination chemistry of these ions is highly similar to that of Mg2+ (Moomaw and Maguire 2008)."***

***This sentence needs to be re-written. I suggest the following: "We conducted a Ni2+ sensitivity assay in E. coli using the N424A-MgtE mutant. Because the the coordination chemistry required for Ni2+ is highly similar to that of Mg2+, MgtE and other Mg2+ channels typically transport Ni2+ (Moomaw and Maguire 2008)."***

We appreciate the comment and corrected the corresponding sentence (From Page 13, Line 15 to Page 14, Line 2).

***Page 13, last paragraph***

***"E. coli cells expressing the N424A mutant of MgtE exhibited increased Ni2+ sensitivity (Fig. 4D) compared to cells expressing wild-type MgtE, suggesting that disruption of the cytoplasmic gate led to increased Ni2+ uptake."***

***This should be moved up and joined with the paragraph above, rather than standing alone.***

We merged this sentence into the previous paragraph, as suggested by the reviewer (Page 14, Lines 6-9).

***Page 14, lines 13-15***

***Furthermore, the TM1 helices that are directly connected to the plug helices have also moved away from the center, as they interact with both the TM2 and TM5 helices (Fig. 5B).***

***Can you add how far these have moved?***

We thank the reviewer for this comment. After checking the corresponding sentence and **Fig. 5B**, we noticed that there was a typo in the text and labels in Fig. 5B. The TM1 and TM5 in the original text and figure should be TM3 and TM4 in the corresponding text and Figure 5B. We apologize for our careless mistakes. We corrected the text and figure and added descriptions of how TM3 and TM4 have moved (Page 15, Lines 5-11) (**new Fig. 5B**).

***Page 15, lines 4-6***

***"Using these mutants with Cu2+ phenanthroline as a catalyst, we performed cross-linking experiments (Fig. 5D and 5E)."***

***I suggest re-writing this to "Using Cu2+ phenanthroline as a catalyst, cross-linking experiments were performed with these mutants (Fig. 5D and 5E)."***

We appreciate the comment and corrected the corresponding sentence (From Page 15, Line 15 to Page 16, Line 1).

***Page 17, lines 7-9***

***"While the expression of wild-type MgtE rescued Mg2+-auxotrophic growth, the mutations at these glycine residues almost abolished Mg2+ transport activity (Fig. 6C)."***

***Comment: Where is the data showing transport activity? If this was not performed, then it should just say growth complementation. Can you show data for bacterial growth?***

As Reviewer #2 mentioned, Fig. 6C shows the growth complementation assay using a Mg^2+^-auxotrophic *E. coli* strain instead of the direct transport assay. Accordingly, we revised the phase of “Mg^2+^ transport activity” to “growth complementation activity” (Page 18, Line 1).

***Page 18, lines 7-10***

***"To be noted, the functional properties of Fab705 would be desirable for determination of the MgtE structure under Mg2+-free conditions because it does not affect the channel gating under Mg2+-free conditions, in particular in an inhibitory manner (Fig. S8)."***

***This sentence does not make sense. Please re-write in a clearer way.***

We appreciate this comment, and we revised the corresponding sentence and added a better explanation of why Fab without inhibitory effects on channel gating would be desirable (From Page 18, Line 14 to Page 19, Line 3).

***Page 18, second to last paragraph.***

***"Based on this structure, we further conducted structure-based functional analysis, and our new findings together with our previous research on MgtE provided structural insights into the gating mechanism of MgtE, as discussed below."***

***This should be joined with the sentence paragraph instead of standing alone.***

We merged this sentence into the previous paragraph (Page 19, Line 1).

***Page 19, lines 3-4***

***"In contrast, under Mg2+-free conditions, the MgtE cytoplasmic domain, including the plug helices, was totally disordered in our cryo-EM map (Fig. 2)"***

***Comment: This is not what Fig.2 shows. Instead you could say that these domains could not be built into the cryo-EM map.***

By following the suggestion from Reviewer #2, we corrected the corresponding sentence (Page 19, Line 13).

***“Page 20, line 7 "1B and 7B)."***

***Comment: The "B)" should be in bold.”***

We appreciate the comment and corrected the corresponding part of the manuscript (Page 20, Line 15).

***Figure legends and figures***

***General comment***

***Helices in legends are labelled as red, but this is not red. You could use salmon.***

According to this comment, we corrected the legend of Figures 1 and 5.

***Figure 1 Legend The "(A)" should be in bold. To the figure you could add arrows to show movement of the N-domain (B) and plug domain (C). Maybe even the distance it has moved could be added to the figure. In (B) and (C) please add more details to the legend, such as which colour refers to what. For (F) and (G) please add more details, such as what the 90 inside the cone means.***

We corrected the legend of Figure 1 and added arrows and angles in Figure 1B and 1C to indicate the structural differences, as we similarly showed in a previous MgtE paper (Hattori *et al*., Nature. 2007, PMID: 17700703, Figure 4).

***Figure 1D and 1E In the methods it states that the ITC experiments were repeated three times with similar results. There are however no details of this in the figure legend. Is the data you are showing an average of these experiments? Or representative? Please include these details in the figure legend. I suggest showing all of the data points for each run, or the error.***

We appreciate the reviewer’s comment. The ITC profiles shown in Fig. 1D and 1E are representative of our three trials. To follow the comment, we stated that they are representative in the legend of Figure 1 and included all ITC profiles and the associated statistics in the new Figure S10 and Table S1.

***Figure 2 Legend Add "Representative cryo-EM image" to "Cryo-EM image". Be more specific for (D-F) with what each is showing. E.g. (D) side views, (E) top-views etc.***

According to this comment, we corrected the legend of Figure 2.

***Figure 2 Label the domains and views.***

To follow this comment, we added labels to Figure 3.

***Figure 3 Label the views.***

According to this comment, we added labels to Figure 3.

***Figure 4 Legend Add PDB IDs where required***

We appreciate this comment. As we mentioned above, we added the PDB IDs throughout the figure legends where required, including the legend of Figure 4.

***Figure 4 You could highlight the residues you mention in the text a different colour. This figure is also showing different structures, then I would use different colour ways for the different structures, and also label them accordingly.***

According to this comment, in the text, we highlighted N332 and N424 in a different color (blue). In addition, we used different colors for the different structures (gray for the Mg^2+^-bound structure in Figure 4B, which is consistent with the Mg^2+^-bound structure in Figure 5) and added labels for the two structures (Mg^2+^-free and Mg^2+^-bound) (Fig. 4A and 4B).

***Figure 5 Legend Add PDB IDs where required. In part D, you need more detail regarding what the figure is showing. E.g. gel. You also say the experiments were repeated six times. Can you show the other gels in supplementary? Is the error in the graph the SEM? If so, state it in the legend.***

We appreciate the reviewer’s comment. According to the comment, we described the PDB ID and stated that error bars represent the standard error of the mean in the figure legend of Figure 5. In addition, we show all six SDS-PAGE gels in the new Figure S11.

***Figure 7 Legend Add "A cartoon representation of the proposed" to "A cartoon of the proposed" Add "The yellow circles containing the letter G" to "The yellow circles containing G"***

We corrected the legend of Figure 7.

***Supplementary***

***Figure S1 Label graphs with conditions, i.e. Mg2+-free, 20 mM Mg2+. It is easier for the reader this way.***

According to this comment, we added labels for Figure S1.

***Can you comment on why the MgtE in magnesium free conditions (orange) is further to the left? This peak elutes in the same position as part A of the figure, with or without Fab so it seems it is to do with the absence of Mg2+?***

We appreciate the comment and apologize for our poor explanation. In gel filtration chromatography, Mg^2+^-free MgtE (Fig. S1A and 1C) eluted earlier than Mg^2+^-bound MgtE (Fig. S1B and 1C). This is probably because of the conformation of MgtE; in particular, the conformation of the cytoplasmic domain is more compact in the presence of Mg^2+^ than in the absence of Mg^2+^, as shown in Figure 1B. We added the corresponding explanation in the legend of Figure S1.

***Page 1, lines 6-9 Part C is not clear. It is helpful for the reader if you refer to the color on the graph in the text. This would be helpful to do throughout the manuscript.***

We appreciate the comment on the legend of Fig. S1. Accordingly, we added more descriptions and labels to refer to each graph and profile for Figure S1. In addition, we referred to the color on the graph in the figure legend of Figure 5E.

***Figure S2 I think it is also helpful here to label the figure with void, Fab-MgtE free-Fab. You could also highlight which area was pooled for cryo-EM data collection.***

We added labels for Figure S2.

***Figure S3 (A) And (B) letters need to be in bold so it is consistent.***

We corrected the legend of Figure S3.

***I also suggest labelling the TMs and the first and last residues in the figure, rather than just the legend. Figure S4 Bold the letters (A), (B) etc.***

We labeled Figure S4 and corrected the legend of Figure S4.

***Figure S5 Lacking details in the legend such as colours.***

We thank the reviewer for this comment. We added more descriptions to the legend of the new Figure S6.

***Figure S6 Bold the letter (A)***

We corrected the legend of the new Figure S7.

***Table S1 I don't think you need to show a row for "With Mg2+ as there is no data, and it has been mentioned in the text.”***

We thank the reviewer for this comment and corrected the corresponding part of the manuscript (Table S1).

***Reviewer #2 (Significance (Required)):***

***“The MgtE family is mechanistically an interesting member as it "blurs" the line between a channel and a transporter. The structure of Mg2+ free MgtE is an important achievement and this structure, in of its own right, will push the field forward. However, if the authors were to invest more time ca. 3 months in developing their model with the current structure (e.g. MD simulations showing Mg2+ conductance, dynamics) it could clarify if and how MgtE functions as a channel? If it doesnt, then perhaps the door should still be left open that this family might be a transporter ...”***

We appreciate the positive comments on the significance of our cryo-EM study (*the structure of Mg^2+^-free MgtE is an important achievement, and this structure, on its own right, will push the field forward*.). However, we respectfully disagree with Reviewer 2’s view that MgtE may be a secondary active transporter, as we addressed above. We think that we caused a misunderstanding; we may have not described the details of the previous patch clamp recordings of the bacterial MgtE and did not properly mention the previous work of SLC41 and similar cases as with the CLC family channels/transporters. As we mentioned above, we added these explanations in the revised manuscript (From Page 5, Line 7 to Page 6, Line 6), and provided further experimental details of our patch clamp recording (**Figure S12**).

Furthermore, to fully respect the request of Reviewer #2, we ran MD simulations over a longer period of time to see if we could observe some structural changes, whereas it is not relevant to determine whether MgtE is a channel or a secondary active transporter. We spent three months running a new MD simulation for 3 μs (**Fig. S8**). The MD simulations of our cryo-EM structure were performed with and without adding Mg^2+^ ion at the Mg^2+^ binding site in the pore. The position of the added Mg^2+^ ion in the pore was based on the previous Mg^2+^-bound MgtE structure (PDB ID: 2ZY9). The cytoplasmic pore remained open in either the presence or the absence of Mg^2+^ ions (**Fig. S8**). Intriguingly, after the 3-μs MD simulation with Mg^2+^ ion, the entrance region of the periplasmic side between the TM1 and TM2 helices became partially opened (**Fig. 8G**), whereas the periplasmic gate still remained non-conductive. To fully understand the gating cycle of the MgtE channel, the structure determination of MgtE in a fully open state is still required. Furthermore, since some of the SLC41 proteins, the eukaryotic counterparts of the bacterial MgtE channel, are Na^+^-driven Mg^2+^ transporters, the understanding of their transport mechanism would require structural analysis of eukaryotic SLC41 proteins. We added the corresponding description in the revised manuscript (From Page 21, Line 15 to Page 22, Line 9).

***Reviewer #3 (Evidence, reproducibility and clarity (Required)):***

***“The study reported by Jin et al. describes the structure and functional analysis of the bacterial magnesium channel MgtE. The authors address an important and outstanding question in the field, which relates to the role of intracellular magneduium in regulating the open/closed state of the channel. The current work reports the structure of MgtE in the absence of Mg2+ ions, revealing a plausible mechanism for regulation. The authors propose a model whereby Mg2+ binding to the cytoplasmic regulatory domain closes the channel by ordering these domains enabling a plug domain to block the pore. The current structure reveals that following Mg2+ dissasocition teh regulatory domain adopts a flexible structure that enables the plug to release and channel to adopt a conducting state.”***

We appreciate the positive comments from Reviewer #3.

***Reviewer #3 (Significance (Required)):***

***“The work is significant for revealing an important state of the MgtE channel and for proposing a plausible model for Mg2+ depedent activation. The paper is well written and the results presented clearly and logically. It would be nice to add some references to similar types of regulation observed in other channels with cytoplasmic regulatory domains, where I understand similar order-disorder transitions have been proposed as regulatory models. The use of FABs is gaining increased traction for determining the structures of small membrane proteins, and I think this study also serves as a good example in this context.”***

We appreciate the positive comments from Reviewer #3. Similar types of regulatory mechanisms, in which the cytoplasmic domains undergo a transition from a rigid conformation to a flexible or disordered state, have been observed in other channels/transporters, such as the CLC Cl^-^ channel (Wang *et al*., PLoS Biol. 2019, PMID: 31022181), the OpuA ABC transporter (Sikkema *et al*., Sci Adv. 2020, PMID: 33208376), which possesses a CBS domain similar to MgtE. We added the corresponding references and discussion to the revised manuscript (From Page 20, Line 15 to Page 21, Line 4).
