# Supplementary material for: The structure of MgtE in the absence of magnesium provides new insights into channel gating
Source: PLoS Biol. 2021 Apr 27;19(4):e3001231. doi: 10.1371/journal.pbio.3001231 (PMC8104411; doi:10.1371/journal.pbio.3001231)
Supplement: Attachment — Submitted filename: Response-To-Reviewers-MgtE-PloSBio-Rev2.docx [file pbio.3001231.s023.docx]

**Response To Reviewers**

***“Based on the reviews (attached below), we will probably accept this manuscript for publication, provided you satisfactorily address the remaining points raised by the reviewers. Please also make sure to address the following data and other policy-related requests:”***

***“a) We ask that you please take into account the concerns of Reviewer #2 and soften the claims on the nature of MgtE as an ion channel in your revised manuscript. Please re-phrase your wording in the discussion section (e.g. ‘the data is most consistent with the protein acting as an ion channel’).”***

We appreciate the comment. As we described the nature of MgtE as an ion channel in the introduction section, not in the discussion section, I assume, the editor meant to ask us to re-phrase our wording regarding the nature of MgtE as an ion channel in the introduction section. Thus, we re-phrased your wording to soften the claims in the introduction section as follows.

*Page 5 Lines 14-15: These results are most consistent with the bacterial MgtE acting as an ion channel.*

In addition, to fully follow the request from the editor, we also added a similar statement in the discussion section (Page 22, Lines 6-7).

***“b) We would like you to consider a suggestion to improve the title as follows:***

***"The structure of MgtE in the absence of magnesium provides new insights into channel gating” “***

To follow this comment, we changed the title (Page 1).

***“Regardless of the method selected, please ensure that you provide the individual numerical values that underlie the summary data displayed in the following figure panels as they are essential for readers to assess your analysis and to reproduce it:***

***Fig. 1D-H; Fig. 2C; Fig. 4C; Fig. 5E; Fig. S2A; Fig. S5B, Fig. S8A-E, Fig. S10A, B and S12 “***

***“*Please also ensure that figure legends in your manuscript include information on WHERE THE UNDERLYING DATA CAN BE FOUND where the underlying data can be found, and ensure your supplemental data file/s has a legend*”***

We provided the individual numerical values that underlie the summary data displayed in these figures (S1 Data). Furthermore, we stated in each of these figure legends that “*The individual numerical values that underlie the summary data displayed in this figure can be found in S1 Data”.*

In addition, while we were preparing the excel file for S1 Data, we noticed that some of the scale bars in Figure S12B are mislabeled due to a typo or simply missing (**Old and New Figure S12**). So, we corrected them (**New Figure S12B**). To be noted, these fixations do not affect any conclusion or statements in the manuscript so that we did not make any other associated changes in the main text.

**Old Figure S12**

**New Figure S12**

***“Please also make the structural data deposited in the PDB (6LBH) and EMD (EMDB-0869) publicly available.”***

The structural data will be automatically released from the Protein Data Bank upon the publication of this manuscript.
